# Immobilization Techniques for Aptamers on Gold Electrodes for the Electrochemical Detection of Proteins: A Review

**DOI:** 10.3390/bios10050045

**Published:** 2020-04-28

**Authors:** Franziska V. Oberhaus, Dieter Frense, Dieter Beckmann

**Affiliations:** Institute for Bioprocessing and Analytical Measurement Techniques, 37308 Heilbad Heiligenstadt, Germany

**Keywords:** electrochemical aptasensor, gold electrodes, protein biomarkers, antifouling strategy, signal amplification

## Abstract

The development of reliable biosensing platforms plays a key role in the detection of proteins in clinically and environmentally derived samples for diagnostics, as well as for process monitoring in biotechnological productions. For this purpose, the biosensor has to be stable and reproducible, and highly sensitive to detect potentially extremely low concentrations and prevent the nonspecific binding of interfering compounds. In this review, we present an overview of recently published (2017–2019) immobilization techniques for aptamers on gold electrodes for the electrochemical detection of proteins. These include the direct immobilization of thiolated aptamers and the utilization of short linkers, streptavidin/biotin interaction, as well as DNA nanostructures and reduced graphene oxide as immobilization platforms. Applied strategies for signal amplification and the prevention of biofouling are additionally discussed, as they play a crucial role in the design of biosensors. While a wide variety of amplification strategies are already available, future investigations should aim to establish suitable antifouling strategies that are compatible with electrochemical measurements. The focus of our review lies on the detailed discussion of the underlying principles and the presentation of utilized chemical protocols in order to provide the reader with promising ideas and profound knowledge of the subject, as well as an update on recent discoveries and achievements.

## 1. Introduction

### 1.1. Aptamers in Biosensing

“Aptamers are set for center stage,” concluded Dr. Rajendrani Mukhopadhyay [1], and, although her statement is 15 years old, it could not be more up-to-date. Today, aptamers have been extensively used in basic research, clinical diagnostics, environmental protection, as well as food safety, and present their promising role as therapeutic tools.

For pathogen recognition, aptamers against bacteria, such as *Escherichia coli*, *Staphylococcus aureus*, and *Mycobacterium tuberculosis*, viruses including herpes simplex virus, hepatitis B and C virus, HIV, and SARS coronavirus, as well as a number of parasites were selected, to just name a few. Aptamers for the recognition of stem cells and cancer, e.g., breast cancer, leukemia, lymphoma, adenocarcinoma, and glioblastoma have been developed, as well as for monitoring environmental contaminations like antibiotics, pesticides, herbicides, and heavy metals toxins. These and other applications were recently listed by Zhang et al. [2]. 

Due to their ability to bind or compete with small molecules and protein ligands, aptamers are considered to be promising therapeutics. They can serve as antagonists to block the interaction of disease-associated targets or as agonists to activate the function of target receptors. Cell-type-specific aptamers can furthermore serve as carriers for the delivery of other therapeutic agents to their intended targets. Numerous efforts have been made on the development of aptamers that can directly act as therapeutic molecules. Ismail et al. [3] and Zhou et al. [4] each offer a recent overview of therapeutic aptamers in the discovery, preclinical, and clinical stages. 

The most prominent application of aptamers is, nevertheless, in biosensing, where the single-stranded nucleic acid molecules acquire a specific three-dimensional conformation in the presence of the target molecule through adaptive folding around the target. The strong binding and specific recognition derives from a combination of geometrical complementarity caused by, e.g., stems, loops, bulges, hairpins, pseudoknots, triplexes, or G-quadruplexes, and molecular interactions, such as electrostatic attraction, van der Waals forces, hydrogen bonds, and pi-stacking of aromatic rings [5]. 

Aptamers exhibit many advantages as recognition elements in biosensing when compared to the traditionally utilized antibodies. Antibodies must be produced biologically by the infection of a substantial number of animals with the desired target molecule, which initiates an immune response. Besides their high manufacturing costs, antibodies are only active and stable under physiological conditions and often suffer variations from batch to batch and short shelf life. Aptamers, on the other hand, convince with their high reproducibility and stability, as well as their low cost.

Perhaps the most important advantage of aptamers is that they are produced in a controlled manner by combinatorial chemistry. From a combinatorial library of 10^15^–10^18^ synthetic nucleic acid molecules, aptamers are isolated via an in vitro iterative process of adsorption, recovery, and amplification known as Systematic Evolution of Ligands by Exponential Enrichment (SELEX). Once the desired sequence is known, the aptamer can easily and quickly be replicated in a DNA synthesizer. RNA aptamers are generally more complicated and expensive to generate, and, although they are more biologically relevant (for example in the form of riboswitches), single-strand DNA aptamers have proven to be just as robust, while their production is cheaper and easier and they possess a higher stability [6]. 

Contrary to antibodies, aptamers can be produced that function under non-physiological conditions (e.g., organic solvents and low pH) by simply running SELEX under the desired conditions. Furthermore, SELEX can be applied to nearly any molecular target, which also includes non-immunogenic and toxic targets. Because aptamers are chemically synthesized, they can be easily modified to increase their life span in the bloodstream, target them to particular locations, or enable their immobilization. Neither modifying nor immobilizing aptamers usually cause a loss in function, which is not the case with antibodies.

Thanks to their unique characteristics and advantages, aptamers are ideal candidates for diagnostic, therapeutic, and (bio)analytical applications. Recent progress in aptamer immobilization on gold electrodes, signal amplification, and the inhibition of biofouling will therefore be discussed in this review. We intend to give an in-depth explanation of the underlying principles and present the utilized chemical protocols in order to provide the reader with profound knowledge of the subject, as well as an update on recent discoveries and achievements.

### 1.2. Principle of Electrochemical Measurements

Besides the recognition element, a biosensor also consists of the transducer, which transforms the signal resulting from the interaction of the analyte with the recognition element into an electric signal. The most common types of transducers used in biosensors include optical, electronic, piezoelectric, gravimetric, and, as discussed in this review, electrochemical transducers. 

Electrochemical biosensing is generally based on a reaction that produces or consumes electrons. As a consequence, either electron transfer across the electric double layer of the electrode takes place that produces a current, or a change in the double layer potential is caused, producing a voltage. There are three main categories of electroanalytical measurement methods: potentiometry, where the difference in electrode potentials is measured; coulometry, where the cell’s current is measured over time; and voltammetry, where the cell’s current is measured while its potential is altered. This subsection aims to provide some background information on redox mediators, as well as cyclic voltammetry and electrochemical impedance spectroscopy, since they are the most frequently used measurement techniques in this review.

#### 1.2.1. Redox Mediators

To enable the desired electrochemical reaction, catalytic labels can be introduced that are either coupled to the target molecule or to the recognition element or subsequently added after target binding in a sandwich assay format. Such catalytic labels include redox-active enzymes, inorganic or organic catalysts or nanoparticles. Label-based sensing is especially beneficial for the detection of low-abundance analytes, but, on the downside, it involves multiple assay steps, requires special reaction conditions, is expensive and time-consuming, and makes real-time measurements impossible.

Alternatively, soluble redox mediators can be used that diffuse to the electrode surface and undergo an electrochemical reduction or oxidation via a heterogeneous electron transfer from or to the electrode, respectively. The driving force for this electrochemical reaction is the energy difference between that of the electrode and the lowest unoccupied molecular orbital (LUMO) of the redox mediator. The energy of the electrode is thereby modulated by the application of a voltage or potential with the use of an external power source. 

**Figure 1:**
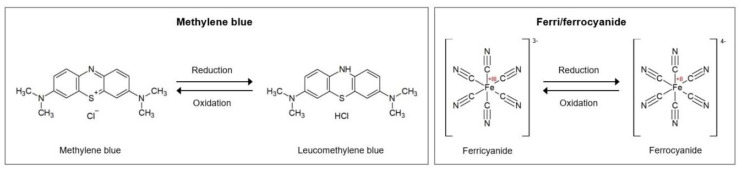
Reduction and oxidation of methylene blue and ferri/ferrocyanide as soluble redox mediators, figures based on the research paper [7].

The diffusion kinetics of the redox mediator to the electrode are influenced by the layers immobilized on its surface. Any change, for example the binding of the analyte, therefore, has an impact on the diffusion efficiency, the electron transfer, and, finally, the detected signal. Depending on the characteristics of the redox mediator, it also interacts with the immobilized aptamers, for which three different modes can be distinguished: (1) Electrostatic interactions of the charged mediator and the negatively charged phosphate groups of the aptamer backbone, (2) groove binding, and (3) intercalation. These mechanisms are discussed in detail in the Section 4.2 The most popular redox mediators are pictured in Figure 1, namely the ferricyanide/ferrocyanide redox couple and methylene blue, which are employed in the overwhelming majority of articles reviewed here.

#### 1.2.2. Cyclic Voltammetry

Cyclic voltammetry (CV) is a powerful and popular electrochemical technique commonly employed to investigate the reduction and oxidation processes. In CV, a voltage is applied that modulates the energy of the electrons in the electrode. When they are at a higher energy level than the LUMO of the utilized redox mediator, an electron from the electrode is transferred to the mediator, that is therefore reduced. Similarly, oxidation can be enforced. The peak potentials, at which reduction and oxidation take place, are characteristic for the individual redox mediators. An overview of the redox potentials of different redox mediators is summarized by Ferapontova et al. [8].

**Figure 2:**
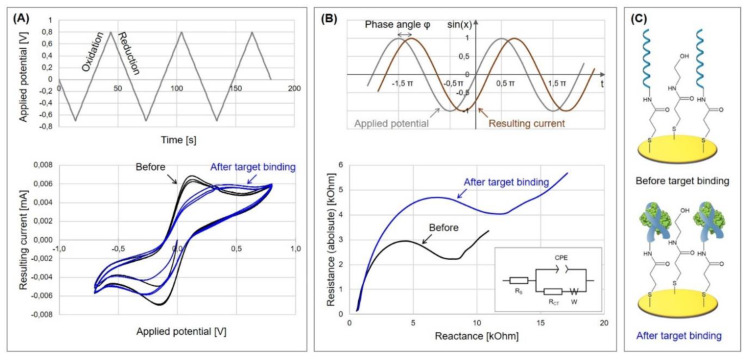
Characterization of target molecule binding to aptasensor via Cyclic Voltammetry (CV) and Electrochemical Impedance Spectroscopy (EIS). Experiments were performed in our laboratory (unpublished results). (**A**) Cyclic voltammogram and applied potential, potential range −0.8 V to +0.7 V with a scan rate of 50 mV/s. (**B**) Nyquist plot of impedance measurement, obtained by scanning in the range of 1 Hz–100 kHz. The inset represents the Randles equivalent circuit. The sine curves represent a general example of applied potential and resulting current in EIS. (**C**) Schematic representation of the utilized aptasensor for the detection of protein A; 3-mercaptopropionic acid was immobilized on the gold electrode, activated with 1-Ethyl-3-(3-dimethylaminopropyl)carbodiimide and N-Hydroxysuccinimide (EDC/NHS) to bind the aminated aptamer, and passivated with ethanolamine.

The applied potential is ramped linearly versus time in as many cycles as needed. Thereby, the rate at which the potential is changed over time is defined as the scan rate. By scanning cathodically to negative potentials, the mediator is reduced by the electrode, which results in a current flow and the depletion of unreduced mediator at the electrode surface. During the reverse scan, the reduced mediator re-oxidizes, resulting in an anionic current. The currents obtained during the forward and backward scans of all cycles are then plotted against the applied voltage to obtain the voltammogram, for which an example is given in Figure 2A. Here, two peaks can be observed that arise from the reduction and the oxidation of the mediator and form the typically obtained “duck” shape. 

But why is there a peak maximum and minimum, if the voltage is continuously increasing or decreasing, respectively? This is due to the fact that the diffusion layer of oxidized (or reduced) mediator continues to grow throughout each scan, which slows down mass transport of unoxidized (or unreduced) species to the electrode surface. Thus, upon scanning to more positive (or negative) potentials, the rate of diffusion to the electrode decreases, resulting in a decrease (or increase) of the measured current as the scan continues. 

The mass transport of the redox mediator is furthermore significantly influenced by the deposited layers on the electrode, by which the recognition element is immobilized. Thus, a number of parameters can be derived from the voltammogram that characterize modification steps and binding events on the biosensor: the anodic and cathodic peak potentials E_p,a_ and E_p,c_; their difference, called peak-to-peak separation ∆E_p_; and the resulting peak currents I_p,a_ and I_p,c_. Binding additional substrates to sensors, either during the fabrication procedure or during target recognition, impedes the diffusion of the redox mediator to the electrode, which leads to decreased charge transfer and resulting current. Therefore, the general tendency can be observed whereby the reduction and oxidation peaks get wider and smaller, resulting in smaller values for peak currents but larger values for peak potentials and their difference. Thus, any binding event at the electrode can be characterized by evaluating the characteristic parameters obtained by cyclic voltammetry. A more detailed practical beginner’s guide to cyclic voltammetry is provided by Elgrishi et al. [9].

#### 1.2.3. Electrochemical Impedance Spectroscopy

Impedance spectroscopy is a powerful method for rapid and label-free analysis of the complex electrical resistance of a system. In the field of biosensors, it is particularly well-suited for the detection of binding events on the electrode, since it is sensitive to surface phenomena and changes of bulk properties. In electrochemical impedance spectroscopy (EIS), a potential perturbation in the form of a sine wave is applied, which induces a current response in the sample under test. The impedance Z of the system is then calculated as the quotient of the applied voltage and the resulting current. It is a complex value of the sum of the system’s resistance R and the reactance X and follows the equation: Z=R−j×X.

The resistance is defined by Ohm’s law and represents the quotient of the applied voltage and the resulting current. Hereby, the resistance is a constant that is independent of voltage and current. If the applied potential, however, varies over time, other effects emerge that can be explained by emergence of the reactance. The reactance is the opposition of the circuit element to a change in the flow of current, which arises from the build-up of an electric or magnetic field. Therefore, the reactance is frequency dependent, while the resistance is not. The response to the potential change is thereby not instantaneous, but causes a lag, which is due to the element’s inductance or capacitance. The charging and discharging of a capacitor, when the potential changes, also causes a lag, so that the current through the element is shifted by π/2 radius relative to the applied voltage. Furthermore, power is not dissipated but partially stored. Therefore, the measured impedance is a complex value, since the resulting current differs according to the amplitude of the applied voltage and shows the phase shift φ, as can be seen in Figure 2B. 

The measured impedance can be pictured either in the form of a Bode plot, where log(│Z│) and φ are plotted as a function of log (f), or in form of a Nyquist plot, which presents the impedance as a vector with the real part (resistance) on the x axis and the imaginary part (reactance) on the y axis. The name impedance “spectroscopy” is derived from the fact that the impedance is generally measured at a series of different frequencies, whereby each measured point pictured in the Nyquist plot derives from the impedance measurement of a period at a single frequency. 

The impedance spectrum allows the characterization of the surface properties and therefore represents the electrical fingerprint of the sample under test. The spectrum is often analyzed by using an equivalent circuit that represents the different physicochemical properties of the system under investigation and is fitted onto the measured Nyquist plot. In biosensing, the plot mostly resembles a semicircle with an affiliated straight line with a 45° slope. It often is fitted with the Randles equivalent circuit (see Figure 2B, inset), which is a model for a semi-infinite diffusion-controlled faradaic reaction to a planar electrode. It consists of the electrolyte resistance R_S_, which results from ion concentration and cell geometry; the Constant phase element CPE, which models the behavior of a double layer at the interface as a capacitance distribution; the charge transfer resistance R_CT_, which refers to the current flow resulting from redox reactions at the electrode surface; and the Warburg impedance W, which results from the impedance at high frequencies due to diffusion from the bulk solution to the interface. An excellent report on modelling elements and their impact on EIS spectra is offered by Bardini et al. [10].

Impedance measurements are often used to follow the different steps of surface modification during the biosensor fabrication. With each step that binds additional components to the working electrode, the diffusion of the redox mediator gets more and more hindered and fewer electron transfer reactions take place, which results in an increase of the charge transfer resistance R_CT_. A decrease can also be obtained, e.g., when aptamers are removed from the sensor in target-induced strand displacement approaches [11,12,13], when redox mediators are utilized that intercalate into dsDNA [12,13], or when gold nanoparticles are bound that have a high electronic conductivity [14]. Thus, any binding event at the electrode can be detected by following the change in R_CT_. 

### 1.3. Formation of Thiol Monolayers on Gold Surfaces

The bioreceptor is immobilized on the electrode in a stable and reproducible manner to enable the reliable detection of the analyte. Here, aptamers again benefit from their small size and versatility to allow efficient immobilization in high-density monolayers, which is of vital importance, especially in miniaturized systems like biosensors. Multiple immobilization techniques have been developed in the past years: (strept)avidin–biotin interactions, electrodeposition, physisorption, and chemisorption, each of which is featured in this review. Since a large quantity of immobilization strategies are based on the chemisorption of thiols to gold, its mechanism and characteristics will be explained in this subsection. Thiols (R–SH), disulfides (R–S–S–R) and sulfides (R–S–R) show a strong adsorption onto metal surfaces, such as gold, silver, platinum, or copper. The sulfur groups spontaneously adsorb from a diluted solution onto the metal surface, forming an ordered and orientated monomolecular layer, which is thus called the self-assembled monolayer (SAM). Gold is, thereby, the substrate of choice because of its inert properties and its formation of well-defined crystal structures, which strongly influence the generation of self-assembled monolayers. The crystallographic orientation of gold, which yields the monolayer with the highest density and degree of regularity, is that of Au(111) [15].

**Figure 3:**
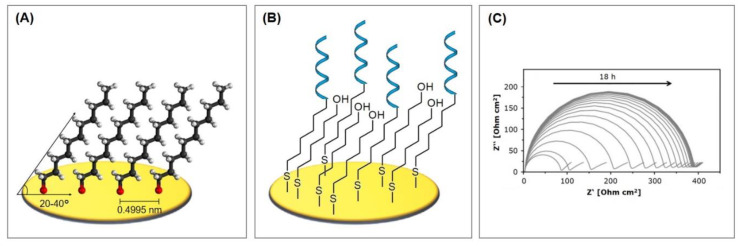
Characteristics of alkanethiol self-assembled monolayers. (**A**) Self-assembled monolayer of alkanethiols, based on [18]. (**B**) Typical biosensor setup consisting of thiolated aptamers and mercaptohexanol (MCH). (**C**) Process of gradual reorganization in alkanethiol monolayers. Xu et al. [30] observed an increasing diameter of the semicircle in the Nyquist plot and therefore a drift of the calculated charge transfer resistance over a period of 18 h after the backfilling with MCH. Reproduced with permission from Xu et al. [30], copyright 2019, American Chemical Society.

The formation of the monolayer follows a three-step mechanism consisting of the diffusion-controlled physisorption, followed by the chemisorption of the molecules, and finally the crystallization process. The physisorbed state on Au(111) can be described as a gas-like, highly disordered system where only van der Waals interactions account for the adsorption. During chemisorption, the sulfur head group loses the mercaptan hydrogen atom (which is believed to generate H_2_) and bonds coordinatively with three gold atoms, forming a strong covalent bond of about 50 kcal/mol [16]. Since sulfur is in a sp^3^-hybridized configuration, the thiol chains tilt from the surface level by 20–40° [17]. In the following crystallization process, the molecules align on the surface in a parallel manner through tail–tail interactions, such as van der Waals, repellent, steric, and electrostatic forces, which results in highly ordered and orientated monomolecular layers [18], as can be seen in Figure 3A.

These monolayers, however, are far from perfect due to several types of defects. A series of strategies can be employed to minimize the defects, but they will always be present to a certain extent. The defects in SAMs can be caused by multiple factors [18]: regions where the molecules have a certain degree of disorder, a small number of missing molecules (also called pinholes), or entire rows of missing molecules that form straight or zig-zag lines. Furthermore, the gold surface itself also shows defects that translate into defects of the monolayer. These include the vacancy island of mono atomic or diatomic depth, steps, and dislocations. 

Factors that remarkably influence the ordering of the monolayer include process parameters like the substrate quality, temperature, choice of solvent, where weaker solvents result in better monolayers [19] and adsorption time. A time of 2–12 h is suitable for long-chain alkanethiols, whereas at least 24 h are necessary for short-chain alkanethiols or thiols with end groups differing from –CH_3_ [18]. The alkyl chain length also influences the SAM order; for n > 10 crystalline configurations are obtained, while shorter chains result in a less ordered and more labile monolayer structure [20]. Lastly, the size of the terminal groups plays an important role. Relatively small groups (e.g., -NH_2_, -H) show no significant influence, whereas bigger groups (e.g., -COOH, ferrocene) reduce the packing density and order of the monolayer [21].

The terminal groups, on the other hand, play an important role in the functionalization of the monolayer. Vericat et al. [18] therefore adequately and nicely describe SAMs as “interfaces between two worlds”, linking metals, semiconductors, and inorganic compounds to organic and biological materials of different complexity. 

## 2. Aptamer Immobilization via Direct Thiolation or Thiolated Short Linkers

A very simple and equally effective method of aptamer immobilization on gold surfaces is their covalent linkage via thiol functionalities. The majority of publications investigate this method in combination with either straightforward approaches, such as backfilling with alkanethiols or dithiols, or more complex strategies for signal amplification or the prevention of biofouling. Furthermore, low-molecular weight linkers, such as cysteamine, 3-mercaptopropionic acid, 3,3′-dithiodipropionic acid, Lomant’s Reagent, aromatic thiols, or trithiaadamantane, are employed that also rely on the formation of self-assembled monolayers of thiolated compounds. Recently published strategies on this topic will be discussed in detail in both this and the following sections. 

A small number of alternatives exist that do not rely on the utilization of thiols. Some of these will also be presented throughout this review and are therefore only briefly referred to here. Since not only sulfur, but also nitrogen, strongly interacts with gold, nitrogen-based functional groups can be used for the immobilization of aptamers. For example, Taghdisi et al. [12] used a poly(thymine) tag that enabled the absorption of capture DNA probes on gold surfaces. Furthermore, gold can be directly modified by electrochemical deposition; Kong et al. [22] grafted aryl diazonium salts onto the gold electrode by performing cyclic voltammetry, while Grabowska et al. [23] and Wang et al. [24] performed the electrodeposition of reduced graphene oxide on gold by the application of a DC voltage. 

### 2.1. Thiolated Aptamers

The overwhelming majority of immobilization strategies rely on the strong interaction of thiol and gold. Mostly, the aptamer is labelled with a thiol group, either directly, or via an alkyl chain or poly(thymine) linker. However, thiolated aptamers do not only bind to gold surfaces via Au–S bonds, but also nonspecifically absorb via multiple nitrogen atoms, which largely restricts the accessibility of the analyte to the aptamer [25]. 

To overcome this issue, Herne and Tarlov [26,27] introduced the backfilling method, in which mercaptohexanol (MCH) is subsequently added to the immobilized aptamers. MCH displaces the nonspecifically adsorbed parts of the aptamers and ensures their upright orientation, owing to the repulsion between the net negative dipole of the MCH alcohol terminus and the negatively charged DNA backbones. Therefore, a well-orientated and organized binary self-assembled monolayer is obtained that results in higher aptamer density, improved accessibility, and lower sample-to-sample variability. These mixed ssDNA/alkanethiol monolayers have been extensively characterized with neutron scattering, X-ray photoelectron spectroscopy, surface plasmon resonance, and electro-chemistry, which have all confirmed the favorable upright orientation of the DNA probes [28].

Keighley et al. [29] later introduced an improved strategy in which aptamers and mercaptohexanol are simultaneously co-immobilized. With the ratio of the substances, the surface density of the DNA can be easily controlled in order to optimize the sensor performance. Thereby, the density should be as high as possible for sensitive detection, while at the same time as low as necessary to allow correct folding and avoid steric hindrance. 

Besides mercaptohexanol, alkanethiols of different length can also be utilized, whereby a number of aspects have to be considered. The length of the linker between the aptamer and its thiol label should be adjusted to allow optimum folding and target binding. Furthermore, the length of the alkanethiol has an impact on charge transfer kinetics, as well as the prevention of nonspecific binding of interfering compounds in clinical or environmental samples (so-called biofouling). Here, the general rule applies: the longer the alkyl chain, the better the antifouling properties (as can also be seen in Figure 8), but also the more insulating the alkanethiol layer. 

For the characterization of impedimetric biosensors that incorporate MCH, it is important to keep in mind that MCH SAMs are thought to undergo a process of gradual reorganization [18,30]. This leads to a thinner but more closely packed layer that exhibits fewer defects, but, at the same time, it is a time-consuming process. Between 2 and 12 h are necessary for long chain alkanethiols, whereas at least 24 h are required for short chain alkanethiols or thiols with end groups differing from –CH_3_ [18]. Within this time frame, an increasing diameter of the semicircle in Nyquist plot, and therefore a drift of the calculated charge transfer resistance, can be observed (see Figure 3C) [30]. This additional time has to be carefully integrated for the characterization of the biosensors in order to avoid obtaining inconsistent readings.

In the last three years, a number of aptasensors that match the criteria of this review have been investigated which utilize MCH or similar alkanethiols. Several of these are discussed throughout this review since they integrate additional strategies for signal amplification or prevention of fouling. Six sensors solely consist of aptamer/alkanethiol and represent the simplest setup of aptasensors. These are listed in Table 1.

The thiol label can be introduced at either the 5′ or the 3′ end, although 5′ is generally preferred. Most of the aptamers are linked to thiol via a C_5_ linker to enable sufficient spacing for aptamer folding and target binding in combination with MCH, a C_6_ alkanethiol. As can be seen from Table 1, the detection limit of the sensors is quite low in a picomolar or femtomolar range for the detection of proteins. The linear ranges cover two to six orders of magnitude. For some of the sensors, the LOD was calculated as 3 × standard deviation of blank/slope of the calibration curve (S/N = 3), which is why it lies within the linear detection range. 

### 2.2. Short Linkers

Besides the direct immobilization of thiolated aptamers, often short linkers are assembled on the electrode via Au–S chemistry, to which the aptamers are subsequently bound. Recent articles that utilize different linkers and the chemistry behind the strategies will be reviewed and discussed in this section.

Ying et al. [37] fabricated an aptasensor for the detection of endotoxin by the deposition of 3-mercaptopropionic acid (MPA) as an intermediate linker, to whose carboxylic group the aminated aptamer was bound using EDC/NHS chemistry (Figure 5). The aptasensor exhibited a linear range of 0.001–1 endotoxin units/mL in impedance spectroscopy and was resistant to nonspecific binding of bovine serum albumin (BSA). 

**Figure 4:**
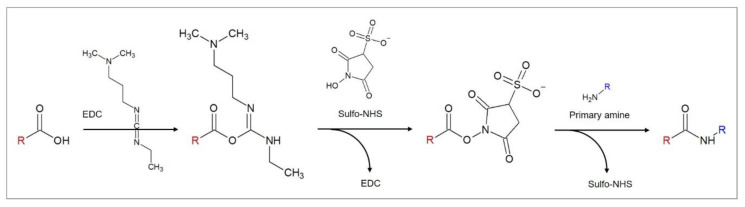
Reaction scheme for the EDC/NHS-assisted amide bond formation between carboxylic acids and primary amines, figure based on [38].

The reaction of carboxylic acids and primary amines is often utilized in aptasensor fabrication. Amide bonds are highly favorable for stable immobilizations due to their poor reactivity that results from the strength of the resonance-stabilized amide C–N bond [39]. The most common reaction strategy for this purpose is a carbodiimide-mediated process using 1-ethyl-3-(-3-dimethylaminopropyl) carbodiimide hydrochloride (EDC) [40], as depicted in Figure 4. EDC reacts with carboxylic acid groups to form an active O–acylisourea intermediate that is, just like EDC itself, water-soluble. O–acylisourea can be easily displaced by nucleophilic attack from primary amino groups, which form an amide bond with the original carboxyl group and release EDC as a byproduct. To improve the yield of the reaction, N-hydroxysuccinimide (NHS) or its water-soluble analogue Sulfo–NHS is used in a second reaction step that leads to the formation of more stable intermediates and allows the conjugation to primary amines at physiologic pH [40]. NHS binds to the carboxylic group, forming an NHS ester and releasing EDC. With the binding of the primary amine, NHS is released and an amide bond between the carboxylic acid and the amide is formed. 

**Figure 5:**
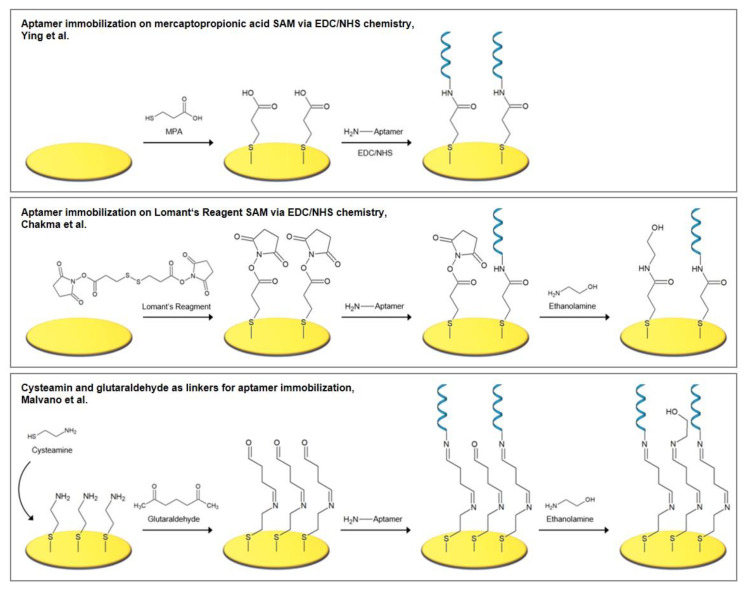
Recently employed short linkers for the immobilization of aminated aptamers [37,41,42].

Chakma et al. [41] investigated a malaria aptasensor for the detection of histidine rich protein II (HRP-II), a specific biomarker for Plasmodium falciparum strains. HRP-II is present in the serum of plasma, cerebrospinal fluid, and urine of infected patients. In the fabrication procedure, dithiobis(succinimidyl propionate) (DSP or Lomant’s Reagent) built a SAM on the gold electrode, onto which an amine-functionalized aptamer was bound. DSP is an amine-reactive cross-linker that carries NHS-ester reactive ends and a disulfide bond in its spacer arm, which cleaves during SAM formation (Figure 5). In the presence of the aminated aptamer or ethanolamine, NHS was released and an amide bond for the immobilization of the aptamer was formed. To passivate the remaining activated carboxylic groups, ethanolamine was subsequently added. The aptasensor had a linear range of 1–500 pM in impedance spectroscopy and showed negligible signal changes when tested with serum.

As an alternative to the conventional EDC/NHS coupling method, an amine coupling process using cysteamine/glutaraldehyde is often employed. Recently, Malvano et al. [42] immobilized an anti-gliadin aptamer (Gli1) on the poly(amidoamine) dendrimer of fourth generation (PAMAM G4) as the aptamer surface anchor. The dendrimer was attached to the gold electrode via cysteamine and glutaraldehyde linkers, that are assembled in a stepwise process. First, cysteamine binds to the electrode via its thiol groups and builds a SAM, onto which glutaraldehyde binds to provide aldehyde groups for the subsequent binding of aminated compounds, here the PAMAM dendrimer. Often, ethanolamine is added in an additional step to passivate the remaining carbonyl groups. The mechanism, which is pictured in Figure 5, is widely employed for the immobilization of numerous types of molecules, such as enzymes [43,44,45], antibodies [46,47,48], DNA [49,50], chitosan [51], and dopamine [52], to name a few.

### 2.3. Drawbacks of Mercaptohexanol

The co-immobilization or backfilling with mercaptohexanol (MCH) or other alkanethiols is currently employed as the standard method. Nevertheless, the utilization of MCH faces a number of serious drawbacks that can result in the irreproducibility of the fabrication, impaired target binding, and nonspecific binding of interfering molecules that inhibit the direct contact with the target analyte and lead to false responses.

As discussed above (Section 2.1) self-assembled monolayers are far from perfect and display several types of defects. Mercaptohexanol has a short alkyl chain length and therefore builds less ordered and more labile monolayers [20] that show significant heterogeneity due to phase domains, multilayers, and pinholes [30]. Moreover, the backfilling with MCH triggers the lateral diffusion of the previously immobilized aptamers that form DNA islands [53,54]. The defects and heterogeneity in the monolayer negatively affect the conformations and adsorption and desorption kinetics of immobilized aptamers and therefore impair target binding [55] and lead to issues regarding reproducibility and long-term stability [54]. 

Another serious challenge which aptasensors that employ alkanethiols have to face is the nonspecific adsorption of interfering compounds of either clinically or environmentally derived samples. These so-called fouling agents include a broad range of biomolecules, such as proteins and nucleic acids, as well as whole cells. Due to incomplete backfilling and defects in MCH monolayers (as well as SAMs of alkanethiols of differing length), remaining bare gold regions have been reported [29,56] to which fouling agents, especially proteins [57,58,59,60], have been reported to bind. 

Although the above-discussed findings on insufficient antifouling properties are not new, co-immobilization or backfilling with mercaptohexanol is still widely employed. We want to emphasize for the reader that this method is by no means the gold standard of immobilization strategies, which can also be seen from the six aptasensors listed in Table 1 that were fabricated in the last three years. Of these, 50% exhibited significant interference from either serum components [32,33] or phospholipids [31] (serum proteins were not tested). Only two of the investigated sensors showed negligible signals from interfering proteins [34,35], while one [36] was not tested for its antifouling properties. 

In this context we also want to refer to two aptasensors discussed below (Section 4.1), that also employ co-immobilization/backfilling with MCH. Here, gold nanoparticles were electrodeposited on the gold electrode in order to increase the electroactive area to obtain a higher aptamer-packing density and therefore an improved sensitivity. While the lysozyme aptasensor [61] showed significant interference from neutravidin and white wine, the aptasensor for interleukin-5 receptor alpha (IL-5RA) [62] demonstrated a drastic increase of the charge transfer resistance when tested with spiked serum.

In conclusion, mercaptohexanol and other alkanethiols cannot sufficiently inhibit biofouling, which represents a serious issue for the application of biosensors because it significantly affects their analytical characteristics including sensitivity, reproducibility, stability, and overall reliability [63]. Therefore, suitable antifouling strategies have to be investigated and employed.

## 3. Antifouling Strategies

Besides the desired specific binding of the analyte, interfering compounds, so-called fouling agents, tend to adhere to the sensor surface. Fouling agents include amino acids, proteins, nucleic acids, phenols, neurotransmitters, and even whole cells or their fragments [54], which nonspecifically bind via a broad range of mechanisms, such as adsorption, polymerization, and precipitation. They either lead to false readings or form an increasingly impermeable layer that inhibits the direct contact with the target analyte. Therefore, biofouling significantly affects the analytical characteristics including sensitivity, reproducibility, stability, and overall reliability [63].

A number of different strategies have previously been investigated to overcome biofouling. However, the majority of these techniques are limited in their application to optical or mass sensitive sensors, since they incorporate high-molecular-weight compounds which are highly disadvantageous for electrochemical transfer reactions [64]. Therefore, only a small number of antifouling strategies exist that are compatible with electrochemical measurements.

In this section, recent advantages in aptasensor fabrication that incorporate antifouling strategies compatible with electrochemical measurements will be reviewed. These include the incorporation of the repellent effect of zwitterionic peptides and the utilization of aromatic thiols that also improve electron transfer, as well as three-dimensional molecules that act as immobilization platforms, such as adamantane molecules, DNA tetrahedrons, and DNA helix bundles. The latter are discussed in Section 4.3.2 and Section 5.2. 

### 3.1. Serum Proteins

For the evaluation of aptasensors that are intended for the detection of biomarkers in serum samples, the proteins that are most abundant in serum should be tested. In doing so, it is essential to investigate these in concentrations that are in suitable relation to that of the biomarker, also taking into account the necessary dilution of serum samples to fit the linear range of the sensor. Therefore, we want to provide the reader with a list of the most abundant proteins in human serum in Table 2. The protein composition in human serum exhibits an uneven distribution: only 22 of the most abundant proteins account for > 99% of total serum proteins [65], which exhibit an extraordinarily high number, namely 10,546 (the latest update of the plasma proteome database [66] was 2014). These include albumin, globulin, immunoglobulins, and lipoproteins. 

### 3.2. Thioaromatic Monolayers

**Figure 6:**
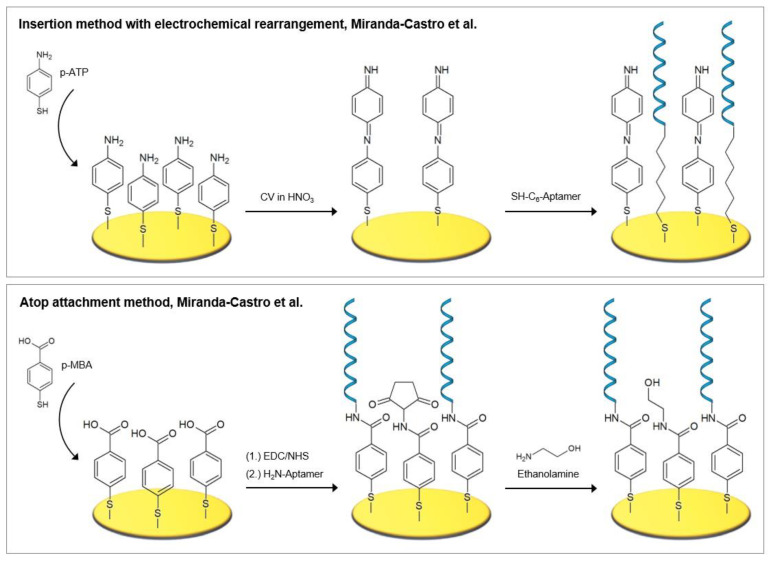
The most successful strategies based on thioaromatic monolayer formation investigated by Miranda-Castro et al. [53].

Self-assembled monolayers of aromatic thiols demonstrate a number of advantages over those of alkanethiols, including improved antifouling properties, packing efficiency, strong structural stiffness, and a higher electrical conductivity compared to alkanethiols, which is due to the delocalized π-electrons in the aromatic phenyl ring [54,70,71,72]. Different strategies for the fabrication of electrochemical biosensors incorporating aromatic thiols, namely p-aminothiophenol (p-ATP) and p-mercaptobenzoic acid (p-MBA), were investigated by Miranda-Castro et al. [53]. These two strategies were the most successful and are also pictured in Figure 6. Firstly, the insertion method with electrochemical rearrangement: the formation of p-ATP SAM, which is altered by potential cycling under acidic conditions, where a N–C_4_ coupling reaction leads to the formation of dimers in a head-to-tail manner. A free gold surface is therefore newly exposed where thiolated DNA is subsequently inserted. Secondly, the atop attachment method: the formation of p-MBA SAM, which is used as scaffold for the covalent immobilization of the amine-modified DNA capture probes. 

With these two strategies, the highest surface density of immobilized DNA probes was obtained, which corresponds to significantly lower limits of specific detection of 16S rRNA of *Legionella pneumophila* or its encoding gene, namely 6 pM for strategy 1 and 40 pM for strategy 2. Most importantly, as the authors stated in a subsequent review [72], the sensors utilizing aromatic thiols demonstrated a remarkable improvement in nonspecific binding, which is expressed in the 6- to 16-fold lower background signal of these sensors compared to the conventional method with MCH. Nevertheless, it has to be noted that the detection of the target molecule was performed in the presence of 2.5% BSA (w/v), which passivated available noncovalent binding sites. The ability to inhibit nonspecific binding of interfering compounds to the sensor itself—without passivation—has therefore to be put into question.

### 3.3. Zwitterionic Peptides

Nonspecific protein adsorption can be minimized by the modification of the gold electrodes with antifouling polymers. Most commonly applied for this purpose are polyethylene glycol (PEG) containing polymers. Zwitterionic polymers, which contain a positively and a negatively charged group in their repeating units, such as phosphorylcholine, sulfobetaine, and carboxybetaine, have recently emerged as superior alternatives. The coexisting repulsive forces and electrostatic attractions between the polymers induce the formation of highly hydrated layers, which are stronger and more s81 than those present in PEG [73,74]. Peptides are natural polymers that are composed of amino acids and therefore inherently zwitterionic. Besides their ease in design and synthesis, peptides possess an outstanding coordination ability and biocompatibility [75], as well as a strong hydrophilicity and neutral net charge, if they are designed for this purpose, that accounts for their remarkable antifouling properties [76]. 

**Figure 7:**
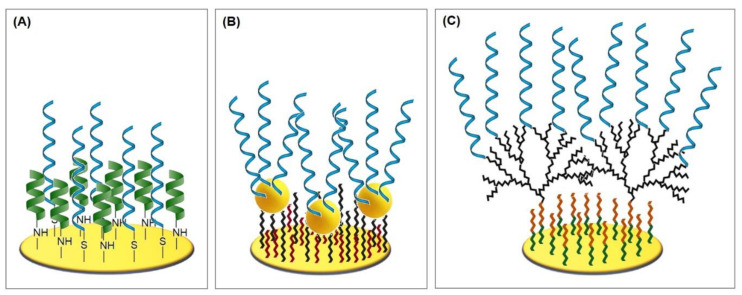
(**A**) Mixed self-assembled monolayer with zwitterionic peptides to inhibit biofouling [77]. (**B**) Gold nanoparticles atop alkanethiol (11-amino-1-undecanethiol) SAM [78]. (**C**) PAMAM dendrimer on cysteamine and glutaraldehyde [42].

Lately, Ciu et al. [77] have designed a new zwitterionic peptide that shows good hydrophilicity and charge neutrality and therefore endows the desired antifouling property to the electrochemical aptasensor for the sensitive and selective detection of alpha-fetoprotein (AFP), a biomarker for liver cancer. In a two-step procedure (see Protocol A1), the thiolated aptamer was immobilized on the gold surface and subsequently backfilled with densely packed zwitterionic peptides of the sequence of EESKSESKSGGGGC. Its C-terminus is amidated and enables the peptide’s adsorption onto the gold electrode (see Figure 7). 

With the binding of AFP, the aptamers undergo conformational changes, which leads to an enhanced resistance to charge transfer in differential pulse voltammetry (DPV) and electrochemical impedance spectroscopy (EIS). The change –ΔI_p_/I_p0_ exhibited linear dependency of the logarithmic value of AFP concentration within the range 10 fg/mL–100 pg/mL in DPV. The exceptionally low limit of detection was calculated to be 3.1 fg/mL. 

The linear detection range is four to six orders of magnitude smaller than the AFP level when it indicates liver tumor (> 500 ng/mL [69]). Therefore, clinical samples have to be severely diluted prior to detection. Since the sensors already exhibited satisfying antifouling ability in 1%–2% serum, the results revealed the potential practical applicability of the investigated aptasensor, modified with zwitterionic antifouling peptides, for the strikingly selective and sensitive detection of the AFP.

## 4. Amplification Techniques

For the detection of low abundance analytes, signal amplification is often necessary. In this section, we want to present recently applied amplification techniques that fit into the framework of this review. Widely employed are strategies based on the increase of the specific surface area that enables the immobilization of a greater number of aptamers. For this purpose, gold nanoparticles are either directly attached to the electrode via electrodeposition or bound to a prior immobilized alkanethiol SAM. Immobilized dendrimers also offer a vast number of functional end groups for the attachment of aptamers. Binding of the redox mediator, either covalently or via intercalation, is also commonly used. Strategies based on the elongation or linkage of the aptamers are discussed, namely hybridization chain reaction, rolling circle amplification, and the newly introduced strategy of target-induced bridge assembly. Finally, the utilization of graphene nanosheets for signal amplification is presented. Some of the findings presented here again highlight the importance of a sufficient antifouling strategy to inhibit nonspecific binding of the interfering compounds present in clinical samples. Here, approaches that only amplify signals obtained from target binding, such as rolling circle amplification, offer a clear advantage. 

### 4.1. Improved Surface Area

The generated signal is generally proportional to probe loading, which is why many amplification techniques aim to increase the specific surface area of the electrode, enabling the immobilization of high amounts of aptamers and the increase in sensor sensitivity. For this purpose, gold nanoparticles (AuNPs) have been widely employed, that can be directly electrodeposited onto the electrode. The gold nanoparticles herein act as nanoscale electrodes that electrically communicate between bioreceptors and bulk electrode materials. If shape directing agents are used during the electrodeposition, shapes other than spheres can be obtained that exhibit an even larger surface area and roughness. As an alternative, dendrimers can be bound to the electrode that enable the direct immobilization of a vast number of aptamers on their surface. 

#### 4.1.1. Spherical and Non-Spherical Gold Nanoparticles

Electrodeposition is generally performed with the use of a simple electrolysis that reduces metal or alloy ions from an aqueous, organic, or fused-salt electrolyte and deposits the uncharged atoms as a thin and tightly adherent coating on the electrode. The metal ion complex thereby chemically adsorbs onto the electrode, followed by electron transfer and reduction of the metal ion that undergoes a crystallization process. With the use of electrodeposition, a broad range of composite materials with unique properties such as abrasion and wear resistance, corrosion protection, and lubricity can be produced [79]. Many experimental factors were found to influence the deposition process, such as current density, bath concentration and pH, and agitation system, as well as time and temperature. These factors must be carefully investigated in order to obtain the optimal properties of the composite material for its desired functions [79]. 

Titoiu et al. [61] lately utilized gold nanoparticles as transducers in the establishment of an electrochemical aptasensor for the detection of lysozyme, an allergenic protein that is used as food additive, e.g., in wine. Gold nanoparticles were deposited using an electrochemical procedure based on pulsed amperometric detection. Compared to the bare electrode, the AuNP modified electrode exhibited higher peak currents and a lower peak-to-peak separation (0.094 V bare vs. 0.080 V modified) in CV measurement as well as a significantly lower charge transfer resistance (99 Ohm bare vs. 29 Ohm modified) calculated from EIS measurement. This emphasizes the increased electroactive area and conductivity of the modified electrode that should lead to higher electrochemical signals and therefore a better sensitivity.

**Figure 8:**
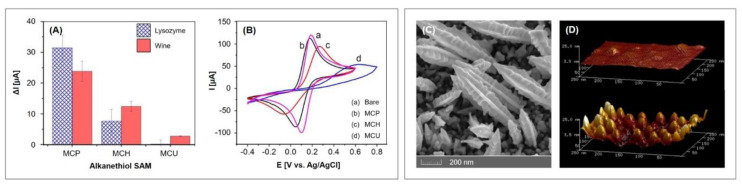
Left: Characterization of 3-mercapto-1-propanol (MCP), 6-mercapto-1-hexanol (MCH), and 11-mercapto-1-undecanol (MCU) monolayers: (**A**) nonspecific binding of lysozyme (10 mg/mL) and wine (diluted 100 times); (**B**) voltammogram. Adapted with permission from the authors of [61], copyright 2019, John Wiley & Sons. Right: (**C**) Field emission scanning electron microscopy image of the fern-leaf-like gold nanostructures, obtained by electrodeposition of AuNPs and PEG 6000. Reproduced with permission from the authors of [80], copyright 2019, Elsevier. (**D**) Atomic force microscopy image of gold electrode before and after AuNPs immobilization onto amino-undecanethiol SAM. Reproduced with permission from the authors of [78], copyright 2017, Elsevier.

Thiolated aptamers with a C_6_ spacer at the 5′ end were immobilized on the AuNP modified electrode and their packing density was evaluated by cyclic voltammetry using [Ru(NH_3_)_6_]^3+^. The obtained average coverage of 3.52 ± 1.316 × 10^12^ molecules/cm^2^ was 2.1 times higher compared to the immobilization on a bare electrode. The backfilling with three alkanethiols of differing length, namely 3-mercapto-1-propanol (MCP), 6-mercapto-1-hexanol (MCH) and 11-mercapto-1-undecanol (MCU), were investigated and MCU was chosen as most suitable since a pure MCU SAM exhibited low nonspecific binding with the investigated wine samples (100-fold diluted) and lysozyme (10 mg/mL), according to Figure 8A. Unfortunately, the long alkanethiol MCU represented a significant barrier for the charge transfer, which resulted in decreased peak currents and a larger peak separation so that the voltammogram lost its typical “duck” shape (see Figure 8B). Furthermore, the resulting SAM was not able to prevent the nonspecific binding of neutravidin (10 µg/mL) and white wine (100-fold diluted) to the aptasensor. The authors also acknowledged that the calculated limit of detection of 0.32 µg/mL and the linear range of 1–10 µg/mL obtained by CV measurements cannot compete with lysozyme aptasensors previously reported.

Youn et al. [62] also investigated an AuNPs modified aptasensor for interleukin-5 receptor alpha (IL-5RA). It was composed of a 5′ thiolated aptamer with a C_10_ spacer that was immobilized on an AuNP modified gold electrode with subsequent backfilling of mercaptohexanol. The results of the AuNP modified electrode’s characterization were in good agreement with the previously discussed findings of Titoiu et al. [61], emphasizing the increase in electroactive area and surface roughness. The aptasensor exhibited a linear range of 10 pg/mL–100 ng/mL for the detection of soluble IL-5RA with a limit of detection of 1.70 pg/mL in electrochemical impedance spectroscopy. Despite these otherwise promising results, the aptasensor showed significant nonspecific binding when tested with serum samples. The charge transfers resistances for the detection of 10 pg/mL–10 ng/mL soluble IL-5RA significantly increased from about 260–700 Ohm in PBS to roughly 4300–6300 Ohm in soluble IL-5RA spiked 10-fold diluted serum (data estimated from graphs).

These publications emphasize two things: on the one hand, the great importance of an effective method to avoid nonspecific binding of interfering compounds of clinical samples; on the other hand, the nevertheless promising basis that the electrodeposition of AuNP forms for a higher aptamer-packing density and increased conductivity due to a higher electroactive area. 

The aforementioned gold nanoparticles were obtained by the electrodeposition of HAuCl_4_ from acidic solutions (6 mM HAuCl_4_ and 100 mM KNO_3_ [62], 1 mM HAuCl_4_ and 0.5 M H_2_SO_4_ [61,81]). If shape directing agents are used during the electrodeposition, shapes other than spheres can be obtained that exhibit a larger surface area and roughness. Negahdary et al. [80] obtained fern-leaf-like nanostructures by the simultaneous electrodeposition of gold and polyethylene glycol 6000. The aptasensor can be used for the detection of amyloid beta (Aβ), a peptide that represent the main component of neurotoxic amyloid plaques found in brains of people with Alzheimer’s disease. Field emission scanning electron microscopy showed rough spindles that greatly enhance the electroactive area (see Figure 8C); the real surface area was calculated to be 0.16 cm^2^, which is 3.5 times higher than of the unmodified electrode (0.045 cm^2^), depicting a high surface roughness factor of 5.1 (unmodified: 1.4).

The aptasensor, fabricated following Protocol A5, exhibited a linear range of 2 pg/mL–1.28 ng/mL and a limit of detection of 0.4 pg/mL in differential pulse voltammetry, and furthermore demonstrated its high reproducibility and repeatability. The regeneration of the aptasensor can be realized by immersion in deionized water for 5 min at 95 °C. Nonspecific binding was tested with hemoglobin, heparin, bilirubin, and HAS, which led to no significant signals at all tested concentrations. Accordingly, the aptasensor demonstrated its excellent selectivity in spiked serum samples (2–200 pg/mL Aß_(1–42)_, serum diluted 1:1 with Tris) with an average recovery of 101.2%. 

#### 4.1.2. Spherical AuNPs on 11-amino-1-undecanethiol SAM

Jolly et al. [78] also utilized spherical gold nanoparticles to amplify the detected signal. Instead of electrodepositing the nanoparticles, they bound the AuNPs onto a previously formed SAM of 11-amino-1-undecanethiol and subsequently co-immobilized the thiolated aptamers and mercaptohexanol on the AuNPs (see Figure 7B and Protocol A3 (Appendix A)). The investigated aptasensor specifically binds prostate specific antigen (PSA), the most commonly used biomarker for prostate cancer detection. Atomic force microscopy revealed the well-ordered assembly of AuNPs on the surface of the electrode that has a roughness factor of 5.56, which is almost nine times higher than that of the unmodified electrode with 0.64 (see Figure 8D). In electrochemical impedance measurement, the aptasensor exhibited a limit of detection of 10 pg/mL and a linear range until 10 ng/mL. The standard binary aptasensor, consisting of aptamers and MCH co-immobilized on a planar gold electrode, only yielded a limit of detection of 60 ng/mL, which emphasizes the significantly improved sensitivity of the aptasensor utilizing AuNPs for higher surface area and probe loading. The sensor’s selectivity was tested with 10 ng/mL human serum albumin, which is six orders of magnitude lower than in serum, although the obtained linear measurement range only allows a maximum dilution by two orders of magnitude. Accordingly, the resulting low signal variations could not be recreated when tested with 10-fold diluted spiked serum samples (recoveries ranging 74.46%–97.64%). 

#### 4.1.3. Dendrimer

Malvano et al. [42] immobilized an anti-gliadin aptamer (Gli1) via cysteamine and glutaraldehyde linker, utilizing the poly(amidoamine) dendrimer of fourth generation (PAMAM G4) as the aptamer surface anchor. The dendrimer has an ethylenediamine core and 64 surface primary amino groups, which react with the 6-carboxylfluorscein label of the aptamer (see Figure 7C and Protocol A6). The advantage of the utilization of PAMAM as an anchor platform over simpler self-assembled monolayer surface coatings lies in the vast increase in the specific surface area of the electrode, enabling the immobilization of high amounts of aptamers and an increase in sensor sensitivity. Moreover, the dendrimer branches maintain their flexibility after the immobilization and therefore expose their functional groups in a more effective way compared to monolayer linkers [82].

Accordingly, the detection limit is one order of magnitude lower when PAMAM is incorporated, compared to the aptasensor without PAMAM, with 5 µg/L and 50 µg/L in electrochemical impedance spectroscopy, respectively. Aptasensors were fabricated with three different PAMAM concentrations (1 mg/mL, 1.5 mg/mL, and 2 mg/mL) that all showed linear behavior in the concentration ranges of 5–50 mg/L and 50–1000 mg/L gliadin. The fabrication process itself displayed a good reproducibility, expressed by relative standard deviations of 4.56%, 5.12%, and 4.25% in five repeats each. After two months’ storage at 4 °C, the aptasensors had a negligible loss of activity, and therefore exhibit an excellent storage stability. 

### 4.2. Binding of the Redox Mediator

In most aptasensors, soluble redox mediators are employed for the quantitative detection of target binding. These assays are cost-effective, simple, and robust and are applicable for the detection of any protein in combination with the appropriate aptamer. The most popular redox indicator—especially for impedimetric measurements—is the ferri/ferrocyanide anion couple due to its sensitivity to surface coverage. Other commonly utilized alternatives are methylene blue and hexaammine ruthenium(III) chloride (Ru(NH_3_)_6_Cl_3_, RuHex) [8].

Depending on the nature of the soluble redox mediator, three different modes of interaction between the mediator and the aptamer can be distinguished. First, the redox mediators electrostatically interact with the negatively charged sugar-phosphate backbone of nucleic acids. To a certain extent, negatively charged mediators are repelled by the aptamer modified surface, which interferes with their diffusion to the electrode surface and therefore partially impedes electron transfer. Positively charged mediators, on the other hand, are attracted, which increases electron transfer. These electrostatic interactions highly depend on ionic strength and pH of the solution [8]. 

Groove binding is the second type of interaction; the mediator binds to the major or minor groove of the DNA double helix by hydrogen bonds and nonpolar interactions involving the methyls and olefinic protons of the pyrimidine bases [83]. The insertion between base pairs represents the third option for mediator/aptamer interaction and is called intercalation. Mediators such as methylene blue, ethidium bromide, and anthraquinone derivatives bind by inserting their planar, aromatic groups in an almost perpendicular position into the double helix axis. Groove binding and intercalation only take place at double-strand DNA and the modes often combine; examples of especially solely groove binding redox mediators are rare [8].

**Figure 9:**
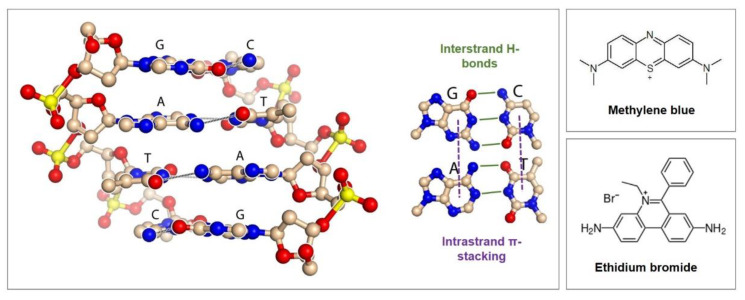
In the DNA helix, the aromatic rings of the bases form π stacks that are responsible for the noncovalent attractive force between the rings. Aromatic redox mediators can intercalate into the helix by inserting their planar, aromatic groups in an almost perpendicular position into the double helix axis. Adapted from the work of the authors of [85].

In aromatic rings, six π electrons are delocalized and can interact with those of another aromatic ring if the rings are favorably positioned. In this process, a positive electrostatic potential on one ring aligns with negative electrostatic potential on another ring to form so-called π stacks that are responsible for the noncovalent attractive force between the rings [84]. Figure 9 shows the organization of the bases in the DNA helix that allow the formation of stacked arrays along the helix axis, followed by the formation of hydrogen bonds across the helix between the complementary bases of the strands. 

Methylene blue is a powerful example of the combination of interaction modes since its positively charged form electrostatically interacts with DNA and its planar aromatic core binds to minor and major groves, as well as intercalates between two successive bases due to favorable π-stacking interactions [8]. Nevertheless, intercalation is the dominant mode of methylene blue interactions with dsDNA [86]. The DNA thereby mediates the charge transport over its π-stacks even over long molecular distances, acting like conductive wires, for which a mixture of tunneling and hopping mechanisms has been proposed [87].

Hexaamineruthenium (RuHex) cations are multivalent, and stoichiometrically bind to the anionic phosphodiester backbone of DNA. Their binding is completely based on electrostatic interactions, because RuHex lacks planar aromatic groups that can intercalate into DNA base pairs. In solutions of low ionic strength, the cations displace charge compensating monovalent cations present at the phosphate backbone [8]. As a result, electron transfer reactions of RuHex are not mediated by the DNA according to the wiring effect, but result solely from the hopping along DNA strands [56].

As an alternative to the above-discussed noncovalent interactions, the redox mediator can be covalently bound to the aptamer. This minimizes false positive readings due to nonspecific binding of interfering compounds and leads to more reliable results [88]. With the utilization of redox-labelled aptasensors, two signaling modes are introduced, depending on the conformation of the aptamer. In the “signal on” mode, the aptamer holds the redox mediator far from the electrode, resulting in no or low signal detection. In the presence of the target, the aptamer undergoes a conformational change that brings the attached redox mediator in close proximity to the electrode surface, resulting in the generation of high electrochemical signals [89]. In the “signal off” mode, however, the signal is switched off due to the target binding induced conformational change of the aptamer. Aptamers forming hairpin structures that hold the redox label close the electrode can be named as an example. When the target binds to the aptamer, the hairpin is opened, which moves the redox mediator away from the surface [88,90]. The previously high electrochemical signal decreases therefore with increasing target binding. 

Cao et al. [13] recently fabricated a sensitive and selective dual-signaling electrochemical aptasensor for the detection of lysozyme, which incorporates the labelling with ferrocene (Fc) and the intercalation of RuHex (see Protocol A7). Lysozymes are considered as inflammatory markers for the diagnosis of several diseases such as sarcoidosis (serum level: 20.71 ± 6.78 µg/mL [91]), as well as chronic myeloid leukemia and myelofibrosis (serum levels: 30–120 µg/mL [92]). Healthy patients have a serum level of 12.93 ± 4.72 µg/mL [91].

The aptasensor consists of two complementary DNA strands, of which one is thiolated at its 5′ end and Fc-labelled on its 3′ end. Eight bases at each end of the probe are complementary and form a stem-loop structure in the absence of the second DNA strand. This strand is the lysozyme aptamer and is neither labelled nor thiolated. The Fc-labelled signaling probe is abbreviated to P-Fc and the lysozyme aptamer to A-Lys. 

**Figure 10:**
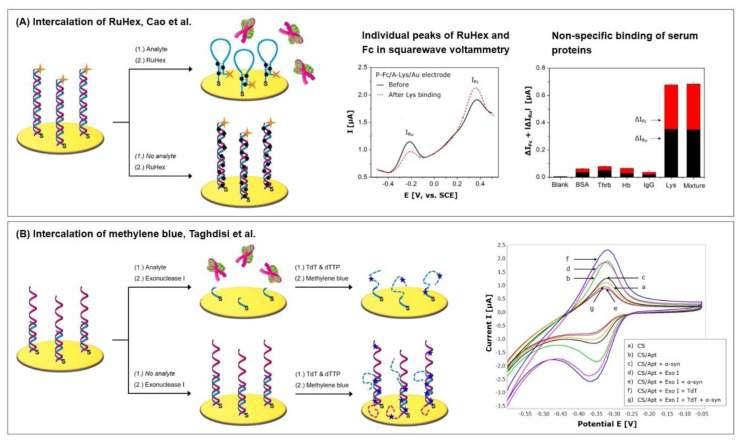
Aptasensors with signal amplification due to redox mediator intercalation and labelling. Graphs are adapted with permission from (**A**) the authors of [13], copyright 2017, Elsevier and (**B**), the authors of [12], copyright 2019, Elsevier.

In the absence of the target lysozyme, as pictured in Figure 10A, the DNA strands are hybridized, holding ferrocene at a distance from the electrode surface due to the dsDNA’s rigid nature, which leads to a weak electrochemical signal of Fc. At the same time, RuHex intercalates into the double strand, resulting in a strong signal. In the presence of lysozyme, A-Lys dissociates from the DNA duplex and binds the target. The remaining P-Fc then forms a hairpin structure, bringing Fc close to the electrode surface, which enhances its signal. The newly formed hairpin structure exhibits a shorter double-strand than the hybridized probes, which is why the RuHex signal decreases with increased target binding.

The superposition of both of the contrary signals of Fc and RuHex is dominated by the Fc “signal on” operating principle; with increasing target concentration, the calculated charge transfer resistance in impedance measurement is decreasing. If square wave voltammetry is applied, two individual peaks appear (Fc at 360 mV, RuHex at –210 mV) that allow the evaluation of the individual signals (see Figure 10A). The superposition of the obtained signal changes ΔI_Fc_ and ΔI_RuHex_ significantly improves the sensitivity and allows to detect in the wider linear range from 10 pM to 100 nM with a limit of detection of 0.8 pM, which equal 143 pg/mL–1.43 µg/mL and 11.44 pg/mL, respectively. 

The specificity of the aptasensor was investigated with 1 µM bovine serum albumin (BSA), thrombin (Thrb), hemoglobin (Hb), and immunoglobulin G (IgG). The results were compared to the signal obtained with 10 nM Lys (Figure 10A) and indicate low interference and high specificity for the detection of Lys. In spiked serum samples (100-fold diluted), recoveries ranging 94.63%–98.66% could be obtained. Taking into consideration that patient serum samples have to be diluted 1000-fold or even 10,000-fold to detect in the low linear range of the sensor, nonspecific binding should be even less, resulting in remarkable antifouling properties. 

Taghdisi et al. [12] also utilized methylene blue intercalation as a signal amplification technique for the highly sensitive detection of the α-synuclein (α-syn) oligomer, an important biomarker related to Parkinson’s and Alzheimer’s diseases. It is based on the target-induced displacement of the aptamer from the immobilized complementary strand and the application of exonuclease and deoxynucleotidyl transferase for the elongation of DNA strands with poly(T). 

Throughout the fabrication process, a short, thiolated single DNA strand that is complementary to the aptamer is immobilized on the gold electrode, to which the aptamer hybridizes, followed by mercaptohexanol backfilling. Two enzymes are subsequently added: Exonuclease I (Exo I), which selectively hydrolyses possibly available ssDNA in 3′-5′ direction; and terminal deoxynucleotidyl transferase (TdT), a DNA polymerase which catalyzes the synthesis of long poly(T) strands at the 3′-OH end of DNA molecules. In the presence of α-syn, as can be seen in Figure 10B, the aptamer binds the target and dissociates from the DNA duplex. The remaining complementary strand is shortened by Exo I activity followed by strand elongation with poly(T) after the incubation with TdT and deoxythymidine triphosphate (dTTP). The subsequently added methylene blue can only interact with the ssDNA by weak electrostatic forces and therefore leads to a low signal response. In the absence of the target, on the other hand, the aptamer and complementary strand remain hybridized with both of the 3′ ends as part of the helix. Exo I therefore does not digest ssDNA, while TdT synthesis creates two poly(T) strands at the duplex. Methylene blue subsequently intercalates in the dsDNA, resulting in a strong current response. 

With the help of Exo I and TdT, a greater difference between the peak currents obtained with and without target (Figure 10B) can be obtained, which results in a higher sensor sensitivity. For the target detection characterization, different α-syn concentrations were directly created in 10-fold diluted serum, instead of buffer. This way, a wide linear range of 60 pM–150 nM could be obtained with a limit of detection of 10 pM. To investigate the repeatability and specificity of the sensor, known concentrations of α-syn were spiked in serum that could be recovered in the range of 95.3%–107.0% with a standard deviation between 1.8% and 4.9%. 

Here, we have to note that, although such a complex amplification strategy was employed, the obtained linear detection range was still too high to examine clinical samples; the phosphorylated form of α-syn can be found in Parkinson’s disease patients with a serum level of 756.8 ng/mL (~ 54 pM), while healthy individuals have a level of 143.4 ng/mL (~ 10.2 pM) [93]. Furthermore, it is strongly advised not to use serum instead of buffer for the measurement of the linear detection range without prior investigation of biofouling, if reliable results want to be obtained.

### 4.3. Linkage or Elongation of the Aptamers

#### 4.3.1. Target-Induced Bridge Assembly

**Figure 11:**
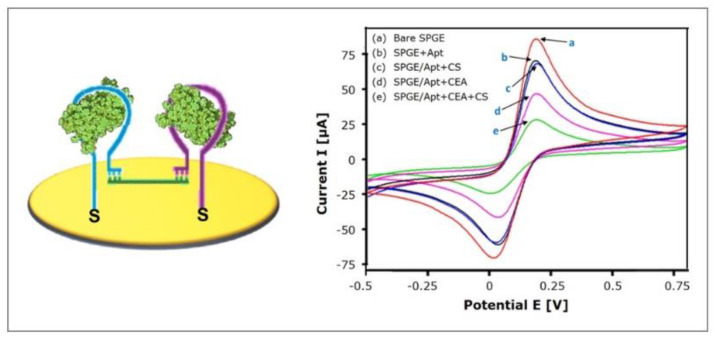
Target-induced bridge assembly: (**A**) Schematic representation of the aptasensor; (**B**) Impact on cyclic voltammetry, adapted with permission from the authors of [94], copyright 2018, John Wiley & Sons.

Taghdisi et al. [94] recently implemented the strategy of target-induced bridge assembly for signal amplification. Two aptamers (Apt1 and Apt2), both specific for the target, are co-immobilized on the gold electrode and form hairpin structures in the absence of the target molecule. The “bridge” to which the strategy owes its name is formed by a ssDNA (CS) that can hybridize with Apt1 and Apt2 using its complementary 5′ end and 3′ end, respectively (see Figure 11). Only in the presence of the target, the hairpin structures are opened, allowing the hybridization with CS, which forms a physical barrier for the diffusion of the redox-mediators to the electrode, therefore decreasing the electrochemical signal.

The investigated electrochemical aptasensor specifically detected carcinoembryonic antigen (CEA), a biomarker for a number of malignant tumors, such as colorectal cancer, pancreatic carcinoma, mamma carcinoma and adenocarcinoma. The sensor performance was investigated using cyclic voltammetry. The linear range was found to be 3 pg/mL–40 ng/mL and the limit of detection was calculated to 0.9 pg/mL. When tested with IgE, thrombin, PSA, HSA, glycine, and myoglobin (40 ng/mL each), no remarkable relative electrochemical response could be detected, indicating the acceptable specificity of the sensor. Subsequently, eight serum samples (20-fold diluted) were spiked with CEA and showed an average recovery of 90.83%–106.06%, emphasizing the interference properties of the aptasensor, and therefore providing reliable readings. 

Taking into account that normal CEA serum levels are ≤ 3 ng/mL [69], patient serum samples could easily be diluted 100-fold, which could further decrease nonspecific binding. On the other hand, the low nonspecific binding, which can be observed—although no antifouling strategy, not even MCH backfilling, was incorporated—might be attributed to the formation of bridges at high target concentrations that shield the gold surface and therefore prevent fouling. In conclusion, the implemented strategy of target-induced bridge assembly for signal amplification enabled the design of a highly sensitive aptasensor with a wide linear range over four orders of magnitude and a remarkably low limit of detection, that shows notable antifouling potential, although no corresponding strategy was applied. 

#### 4.3.2. Amplification via Hybridization Chain Reaction

Dirks et al. [95] introduced the method of hybridization chain reaction (HCR) that functionalizes DNA as an amplifying transducer for biosensing applications. This simple isothermal enzyme-free amplification method combines the advantages of high versatility and simplicity and is regarded as an attractive technique for DNA nanotechnology, biosensing, bioimaging, and biomedicine. Therefore, a number of electrochemical aptasensors that integrate HCR have been investigated for the detection of arsenic [96] and mercury [97], adenosine triphosphate [98], 8-hydroxy-2′-deoxyguanosine (biomarker for oxidative stress) [99], tetracycline [100], kanamycin [101], lysozyme [102], and *Escherichia coli* O111 [103], and for monitoring the protein kinase A activity [104].

**Figure 12:**
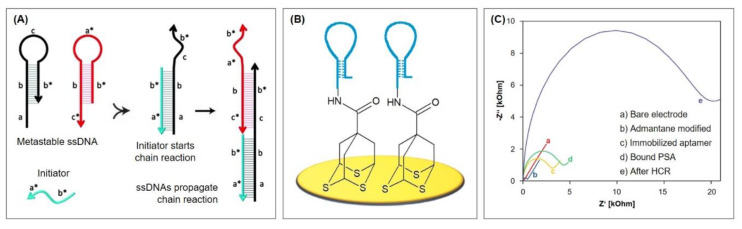
Hybridization chain reaction: (**A**) Mechanism; (**B**) Aptasensor with adamantane as linker for the aptamer [105]; (**C**) HCR impact on detected EIS signal; adapted with permission from the authors of [105], copyright 2018, Elsevier.

The key to HCR is the storage of potential energy in two ssDNA hairpin species H1 and H2 that consist of short loops protected by long stems [95]. Therefore, the hairpins are stable and coexist in solution until a single-stranded DNA initiator is added, which triggers a cascade of hybridization events (see Figure 12A). The initiator strand opens and binds the complementary region of the first hairpin species H1, which then exposes a new single-stranded region that opens the hairpin of the other species H2. This exposes a single-stranded region identical to the sequence of the initiator and can therefore bind H1. The chain reaction of hybridization events between alternating H1 and H2 hairpins forms a nicked double helix that grows until the hairpin supply is exhausted. 

Recently, Ding et al. [105] developed an impedimetric aptasensor for the detection of prostate specific antigen (PSA) by immobilizing 7-hydroxycarbonyl-2,4,9-trithiaadamantane for the first time on a gold electrode via gold-thiol chemistry. This three-dimensional adamantane molecule acts as a stable tripodal surface anchor for the aptamer and therefore enables its optimized, active orientation, which promises efficient protein recognition. This way, a well-aligned DNA monolayer is achieved, whose density can easily be controlled by adjusting the adamantane concentration. The three thiol vertices facilitate a strong binding of the complex whose immobilization is completed after just 4 h. 

The top carboxyl group of adamantane is activated in order to bind the aminated aptamer, whose 3′ end resembles the initiator for the HCR after binding of PSA (see Figure 12B and Protocol A8). Due to its negatively charged backbone, the long double-stranded DNA resulting from HCR absorbs a large quantity of the redox mediator RuHex. Therefore, the impedance increases remarkably (see Figure 12C), which emphasizes the excellent signal amplification properties of the system. Accordingly, the calculated limit of detection is notably low with 0.05 pg/mL, obtained by chronocoulometry. The aptasensor is protein-resistant and does not require backfilling with, e.g., alkanethiols. This was tested with high concentrations of alpha fetoprotein (AFP), carcinoembryonic antigen (CEA), and human chorionic gonadotropin (HCG), whose signals were similar to the background signal. Therefore, the following PSA detection in serum samples of five patients was in good agreement with those obtained by an immunoradiometric method. The sensor also demonstrated excellent reproducibility: the procedure was performed on five independent electrodes and yielded a relative standard deviation of 4.63%. 

#### 4.3.3. Rolling Circle Amplification

Rolling circle amplification (RCA) is an isothermal DNA amplification technique that, due to its versatility, serves as an attractive tool for biomedical research and nanobiotechnology. It mimics the naturally occurring replication process of circular plasmids and viral genomes, including those of bacteriophages, eukaryotic viruses, and circular RNA genomes of viroids, as well as of extrachromosomal DNAs in amphibians. 

**Figure 13:**
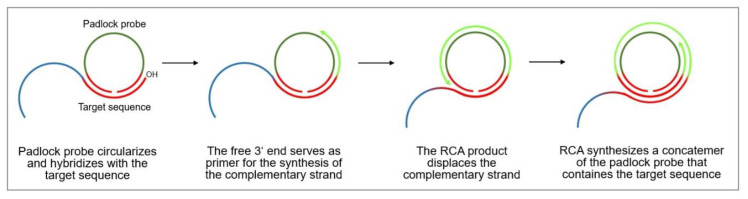
Schematic representation of the mechanism of rolling circle amplification, based on the research of [108].

The rolling circle DNA replication [106,107] starts with the cleavage of one strand at a site called the double-strand origin, producing a 3′-OH and 5′-phosphate terminus. The 3′ end serves as the primer for the subsequent DNA synthesis that uses the unnicked strand as a template. As the replication proceeds around the circular template, the 5′ end is displaced, unrolls from the circular template and increases in length (see Figure 13). Since the circular DNA template can be replicated many times, rolling-circle replication produces multiple single-stranded linear copies that are attached head-to-tail and are called concatemers. These linear copies can be converted to double-stranded circular molecules by the site-specific cleavage of the single-stranded tails at the origins of replication, synthesis of the complementary strand and recirculation. 

Rolling circle amplification can therefore be used to generate a long single-stranded DNA concatemer of the original DNA target [108]. As template for RCA, a ssDNA minicircle, the so-called padlock probe, is used that hybridizes to the target sequence. The free 3′ end of the target sequence is the primer for RCA that enables the synthesis of linear replica of the padlock probe until the process is terminated. This way, a more than billion-fold amplification can be achieved at a constant temperature (room temperature to 37 °C) within 1–2 h, making RCA a powerful and simple tool for the ultrasensitive replication of specific DNA sequences [109]. Moreover, the technique is highly versatile, since the primers and padlock probes can be designed according to one’s needs, allowing to finely tune the length, sequence, composition, structure, and rigidity of the RCA product. It can also introduce functional sequences including DNA aptamers, DNAzymes, spacer domains, and restriction enzyme sites, as well as functional moieties like fluorophores, biotin, antibodies, and nanoparticles. 

**Figure 14:**
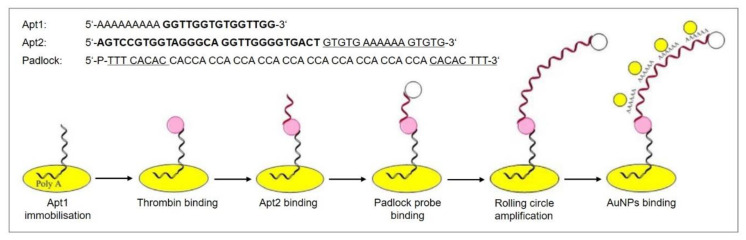
Stepwise fabrication of an aptasensor incorporating rolling circle amplification and gold nanoparticle binding for signal amplification. The DNA sequences are shown on top: Thrombin binding sites are pictured in bold, complementary bases underlined. Adapted with permission from the authors of [14], copyright 2018, Elsevier.

Fan et al. [14] recently introduced blocks of multiple adenosine nucleotides (polyA blocks) to their RCA product, which was thus able to selectively and strongly bind gold nanoparticles (see Figure 14). The AuNPs electro-catalyze the reduction of H_2_O_2_, and, therefore, a dual signal amplification in a sandwich format is obtained. The aptasensor detects thrombin, a serine protease that plays a significant role in blood coagulation, which is a biomarker for the diagnosis of a number of diseases, such as pulmonary metastases and diseases associated with coagulation abnormalities. 

**Figure 15:**
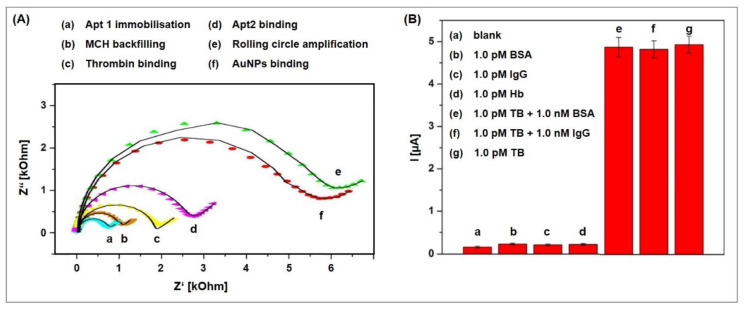
Rolling circle amplification integrated in thrombin aptasensor. (**A**) Nyquist plots of the stepwise aptasensor fabrication; (**B**) Examination of selectivity (corrected version). Adapted with permission from the authors of [14], copyright 2018, Elsevier.

The ssDNA resulting from rolling circle amplification was characterized using atomic force microscopy. Long and curved DNA nanostructures of 2 µm length could be observed with well-separated AuNPs along the skeleton. Impedance spectra were recorded to study the stepwise assembly of the aptasensor, which are shown in Figure 15A. With each step, starting from Apt1 immobilization (a) to Apt2 binding (d), the charge transfer resistance increased, indicating the formation of a layer that more and more repels and hinders the diffusion of the redox mediator. After rolling circle amplification (e), the charge transfer resistance dramatically increases, pointing at the striking impact that the newly formed long ssDNA have on the electrochemical behavior of the aptasensor. The subsequently attached AuNPs (f) lead to a decrease resistance due to the high electronic conductivity of AuNPs. 

The aptasensor showed linear behavior for the detection of thrombin over the range 100 fM–10 nM and a remarkably low limit of detection of 35 fM in differential pulse voltammetry. The exceptional sensitivity of the proposed sensing platform is caused by the fact that a single bound thrombin molecule generates a long DNA strand that binds many AuNPs, which significantly enhance the signal. To investigate the impact of the RCA product on the sensitivity, the aptasensor was also fabricated without RCA, where the AuNPs directly adsorb on Apt2. The limit of detection was 5.6 pM and therefore 160 times higher, which emphasizes the advantage of RCA utilization for signal amplification.

Nonspecific binding was tested with bovine serum albumin and immunoglobulin G and led to only negligible electrochemical responses (see Figure 15B). The subsequent spiking of thrombin into 10-fold diluted serum led to recoveries ranging 97.6% to 105.4% and RSD ranging 3.8% to 6.4%. These findings highlight the advantage of sandwich approaches for signal amplification since only signals obtained from target binding are amplified, whereas false-positive signals of nonspecifically bound interfering substances are not enhanced, leading to highly reliable and sensitive signals.

### 4.4. Graphene Nanosheets

**Figure 16:**
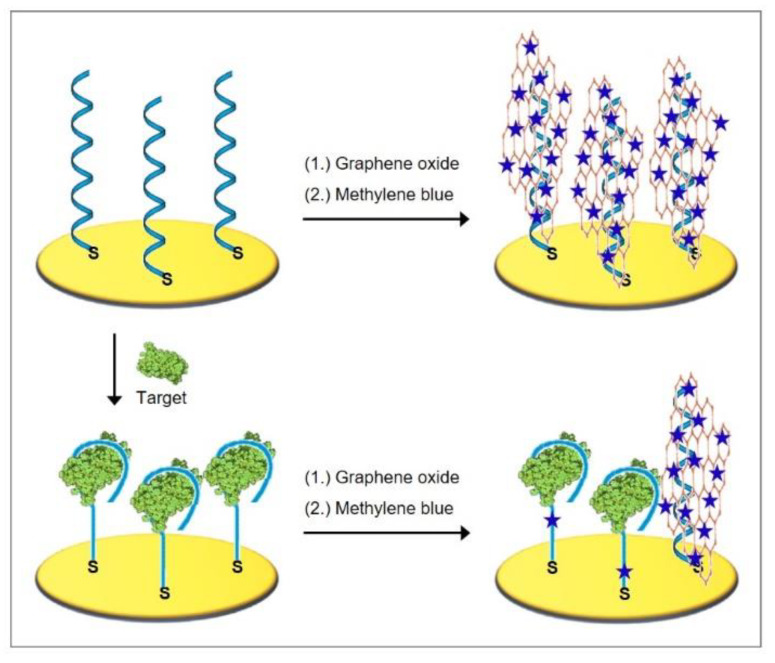
Schematic representation of signal amplification via graphene oxide nanosheets, investigated by Peng et al. [110].

Graphene nanosheets can also be utilized for signal amplification, for example when they interact with previously bound analytes or with aptamers that remain free after analyte incubation. The only recent article employing this strategy was published by Peng et al. [110], who fabricated an aptasensor for the detection of *Cronobacter sakazakii*, which accounts for a number of extremely high-mortality diseases, such as sepsis and meningitis. The aptasensor was assembled by the immobilization of thiolated aptamers and backfilling with mercaptohexanol. The signal of bound target bacteria was amplified by the subsequent addition of GO nanosheets that strongly interact with free aptamers via π–π stacking (see Figure 16 and Protocol A2). A large amount of methylene blue adsorbs to GO by electrostatic interaction, which enhances the signal. The fewer bacteria bind, the more GO and methylene blue is bound to the sensor, resulting in higher signals in differential pulse voltammetry. This way, a wide linear range of 2 × 10^1^ to 2 × 10^6^ CFU/mL and a detection limit of 7 CFU/mL are obtained. As an alternative to DPV, impedance spectroscopy could be utilized, since bound GO/MB results in an impedance decrease; the fewer bacteria bind, the lower the impedance, which is further decreased by GO/MB binding to the remaining aptamers. 

The aptasensor demonstrated its excellent specificity when tested with seven foodborne pathogens, among them *Salmonella typhimurium, Escherichia coli*, and *Staphylococcus aureus*, since no significant signals were detected. With spiked serum samples, recoveries of 96.2%–106.2% could be obtained, emphasizing the sensor’s reliability, as well its remarkable antifouling properties. 

As an alternative to the only recently published article, we want to present two additional strategies that exploit graphene oxide nanosheets for signal amplification in electrochemical aptasensors. We aim to highlight their versatile usability as well as their inherent antifouling properties, which result from the sandwich-like approach that only amplifies signals derived from the sensor’s interaction with the analyte, while signals of possible fouling remain unamplified. 

**Figure 17:**
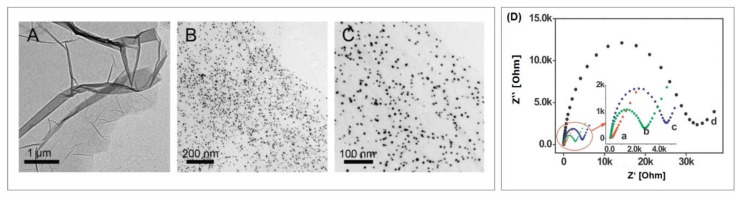
(**A**) High-resolution transmission electron microscopy (HRTEM) image of graphene oxide nanosheets; (**B**,**C**) TEM images of reduced graphene oxide nanosheets modified with gold nanoparticles. Reproduced with permission from the authors of [111], copyright 2015, Elsevier. (**D**) EIS response of (**a**) bare, (**b**) capture DNA, (**c**) aptamer hybridized to capture DNA, (**d**) rGO/AuNP/DNA2 bound to the aptamer, reproduced with permission from the authors of [11], copyright 2014, Elsevier.

**Figure 18:**
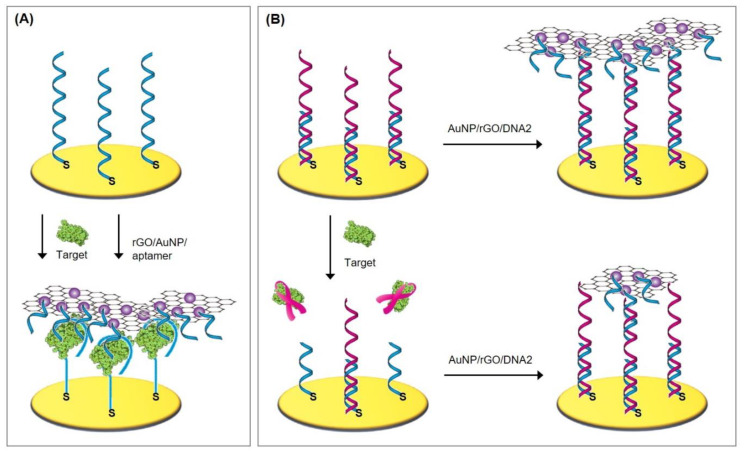
Signal amplification strategies based on graphene nanosheets, employed by (**A**) Wang et al. [111] and (**B**) Jiang et al. [11].

Wang et al. [111] investigated a sandwich sensing platform for signal amplification, in which an aptamer functionalized graphene oxide nanosheet was bound to the analyte after its binding to the aptasensor (see Figure 18A). This way, the rGO/AuNP/aptamer nanocomposite shielded the sensor surface and accounted for a dramatic impedance increase. In the fabrication process, thrombin binding aptamer TBA15 and dithiothreitol were co-immobilized on the gold electrode, followed by incubation with the analyte thrombin. The composite of graphene oxide nanosheets and gold nanoparticles (see Figure 17A–C) was synthesized in a one-pot reaction according to literature [112] and functionalized with the second aptamer TBA29 that had a different binding site than TBA15. Rhodamine 6G was added to block the remaining space on the AuNPs to inhibit nonspecific binding of serum proteins. The aptasensor has a linear range of 0.3 nM to 50 nM with a detection limit of 10 µM. The measured impedance tremendously increased with thrombin concentration, so that with 50 nM analyte, a charge transfer resistance of about 30,000 Ohm was reached. Since a sandwich format was employed that only amplifies signals generated by target binding, the sensor exhibited excellent antifouling properties. 

Jiang et al. [11] utilized an amplification technique that was based on target-induced strand displacement. Similarly to Wang et al. [111], a graphene oxide/gold nanoparticle composite was synthesized, to which thiolated reporter DNA was immobilized. The protocols for the synthesis of rGO [113] and the immobilization of reporter DNA [114] were taken from the literature. The sensing interface consisted of thiolated capture DNA and MCH that were immobilized on the gold electrode, while the aptamer was bound via hybridization to the capture probe. In the absence of the analyte ochratoxin (OTA), one of the most toxic foodborne mycotoxins, the rGO/AuNP/DNA2 composite binds to the aptamers via DNA hybridization (see Figure 18B). The bound composite heavily impeded redox mediator diffusion, which therefore led to a dramatic impedance increase, as can be seen in Figure 17D. In the presence of OTA, the aptamer/OTA complex dissociates from the capture DNA. This resulted in fewer aptamers present for rGO/AuNP/DNA2 binding, and hence a lower signal. 

From TEM images, the authors were able to conclude that a large number of AuNP with an average diameter of 15 nm were homogeneously and densely distributed on the rGO sheet. Due to the fact that each AuNP can be loaded with a couple of hundred reporter DNAs, the charge transfer resistance of the biosensor is increased at least sevenfold by rGO/AuNP/DNA2 binding. This results in a remarkably low detection limit of 0.3 pg/mL and a linear range of 1 pg/mL–50 ng/mL. Incubation with fumonisin B1 and ochratoxin B did not lead to signal change, demonstrating the sensor’s high specificity. Nevertheless, interference due to nonspecific binding cannot be ruled out, since the aptasensor exhibited recoveries ranging 90%–97% when tested with spiked red wine samples.

## 5. Immobilization via Streptavidin/biotin Interactions, DNA Nanostructures, as well as Reduced Graphene Oxide and Pyrene

### 5.1. Immobilization via Streptavidin/avidin Interaction with Biotin

Streptavidin is a tetrameric protein that is produced by the actinobacterium *Streptomyces avidinii*. Avidin, on the other hand, is a protein present in the egg white of birds, reptiles, and amphibians. Both proteins consist of four identical subunits that each can bind to the vitamin biotin with an unusually high affinity to form one of the strongest noncovalent bonds known. 

**Figure 19:**
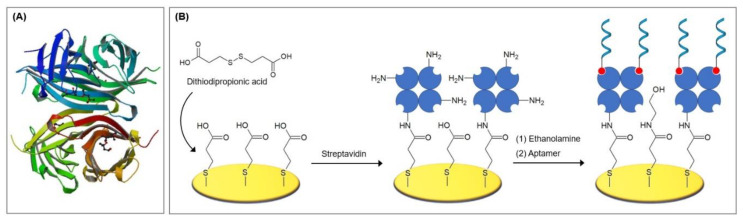
(**A**) Tetrameric streptavidin binding biotin molecules, reprinted from the paper [118]. (**B**) Human osteopontin aptasensor utilizing the streptavidin/biotin interaction [119,120].

Weber et al. [115] were the first to investigate the structural organization of the streptavidin subunit that consists of 159 amino acids. These are organized as eight-stranded, anti-parallel beta sheets that fold into a barrel tertiary structure with several extended hairpin loops (see Figure 19A). Four identical subunits associate to form the tetrameric quaternary structure, which is stabilized by extensive van der Walls interactions. Livnah et al. [116] found out that the overall folding and the structure of the binding pocket of streptavidin and avidin closely resemble one another. There are only two major differences between the binding pocket of avidin compared to streptavidin, namely the presence of an additional aromatic group and a larger number of hydrogen bonds to biotin. This results in a tighter binding of biotin to avidin, which is reflected by the smaller dissociation constant K_D_ = 6 × 10^−16^ M compared to streptavidin with 4 × 10^−14^ M [117]. 

Recently, Meirinho et al. [119,120] developed an aptasensor for the detection of human osteopontin (OPN), a potential breast cancer biomarker. The aptamer was selected using the SELEX process and exhibited a satisfactory affinity towards OPN with a dissociation constant lower than 2.5 × 10^−9^ M. The biotinylated aptamer was immobilized on gold electrode following Protocol A9. In this procedure, 3,3′-dithiodipropionic acid forms a self-assembled monolayer, whose carboxylic groups are activated by EDC and NHS in order to bind the free amine group on the N-terminus of streptavidin (see Figure 19B). The remaining active carboxyl groups are blocked by ethanolamine. Subsequently, the aptamer is immobilized via its 5′-biotin tag to create an aptasensor with a linear range of 25–100 nM and a calculated limit of detection of 1.4 ± 0.4 nM in square wave voltammetry. The sensor demonstrated good reproducibility and acceptable selectivity, exhibiting low signal interferences from other proteins (THR, BSA, rbOPN and LYS). In spiked serum samples, the aptasensor quantified similar OPN levels (2.2 ± 0.7 nM, ~141 ng/mL) compared to the standard enzyme-linked immunosorbent assay (ELISA) method (1.7 ± 0.1 nM, ~108 ng/mL).

### 5.2. Immobilization via three-dimensional DNA nanostructures

By immobilizing aptamers on electrode surfaces, heterogeneous systems are established that exhibit a reduced accessibility of the analyte to the immobilized probe compared to the probe-target recognition in homogeneous solutions [26,121,122,123,124]. Furthermore, entanglement between probes and local aggregation in self-assembled monolayers are often encountered as possible complications when aptamers are directly immobilized on a surface [125]. It is, therefore, critically important to precisely control aptamer density and orientation to minimize interactions between probes and to maximize target accessibility [126].

For this purpose, Pei et al. [127] developed a new concept to significantly enhance the spatial positioning range and the accessibility of the DNA probes by utilizing three-dimensional DNA nanostructures as anchoring points for the bioreceptor. The structures resembled tetrahedrons and consisted of three thiolated ssDNA fragments and one probe containing ssDNA fragment that positions the probe on the tetrahedron’s top vertex (see Figure 21A). Assembly is fast and simple to perform by mixing stoichiometric equivalents in buffer, heating, and then rapidly cooling to 4 °C. Within two minutes, the tetrahedron assembly process itself is completed. As a model system, horseradish peroxidase was bound to the tetrahedron that functioned as signal transduction via catalytic reduction of peroxide. The enzyme was avidin-labelled and therefore could bind to the biotinylated target DNA, that is complementary to the immobilized probe on the top vertex. Even though no rigorous signal amplification was integrated into the system, the limit of detection for the target DNA was 1 pM and thus about 250-fold more sensitive compared to a ssDNA probe/MCH-based sensor with a similar HRP-based signal transduction [128].

This three-dimensional DNA nanostructure serving as an aptamer anchor demonstrates a series of advantages over the direct immobilization of aptamers on the surface. The tetrahedrons have a high mechanical rigidity and organize the aptamers in a highly ordered, upright orientation, which avoids interprobe entanglement [126,129] and grants superior molecular accessibility [130,131,132]. The probes on the nanostructure interface are placed in a solution-phase-like environment, where diffusion and convection are expected to be higher than in macroscopic ones [126]. Since one tetrahedron structure is immobilized via three thiol groups, the stability of the immobilized probes is greatly enhanced (approximately by 5000 times) when compared to mono-thiolated DNA. Furthermore, DNA nanostructures prevent nonspecific adsorption of proteins without the need for backfilling with alkanethiols. Tetrahedron-modified surfaces are versatile platforms that enable various functionalizations with nucleic acids for biodetection, small molecules, antibodies, and nanoparticles [133], and have therefore been utilized for the detection of a broad range of molecules and structures, such as Hg^2+^ ions [134], cocaine [135], microRNA [131,136,137], cancerous exosomes [138], and cancer cells [139]. 

**Figure 20:**
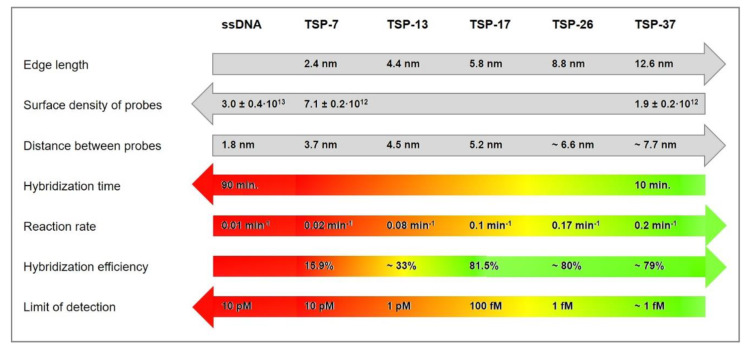
Characterization of DNA tetrahedrons of different sizes (the numbers refer to the base pairs of each edge of the tetrahedron) and comparison to directly immobilized ssDNA probe. Data taken from the authors of [140], estimated data (~) extracted from graphs. The color gradient intends to give a simple overview of general tendencies, whereby the red color was by no means meant to signify poor results.

Each aptamer is anchored to an individual tetrahedron and therefore the aptamer distance and interaction are finely tuned by the tetrahedron size. Lin et al. [140] produced five tetrahedron types (TSP-7, TSP-13, TSP-17, TSP-26, TSP-37, numbers refer to the base pairs of each edge of the tetrahedron) to investigate the influence of their size on DNA surface hybridization and sensor performance. They found that with the increase in tetrahedron size and therefore probe distance, the hybridization kinetics, as well as sensitivity of the sensor, improved significantly. In order to study the limit of detection, a standard sandwich assay for the detection of target DNA, consisting of biotinylated reporter probe and avidin-HRP, was carried out with cyclic voltammetry. The obtained results and general tendencies are lucidly summarized in Figure 20. It can be concluded that, overall, more satisfying results are obtained with tetrahedrons of a greater edge length.

**Figure 21:**
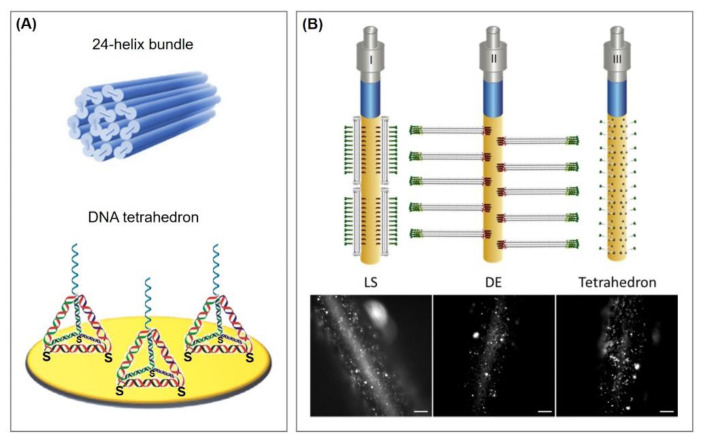
(**A**) Investigated 3D DNA nanostructures; (**B**) Schematic overview of nanostructure binding to the gold surface of FO-SPR sensor tips. The bundles were modified with ssDNA at either the lateral surface (LS) or the distal ends (DE). The ssDNA served as linkers to the gold surface (red representation) or as anchor points for aptamer immobilization (green representation). Below, fluorescence microscopy images of fluorescence labelled ssDNA hybridized to immobilized aptamers. Scale bars represent 100 µm. Adapted with permission from the authors of [141], copyright 2018, American Chemical Society.

Daems et al. [141] recently compared tetrahedrons (Protocol A4) to rigid 24-helix bundles (Protocol A10) as an alternative platform for aptamer immobilization. They synthesized 24-helix bundles—similar to a previous report [142]—that were modified with several ssDNA strands, which either served as linkers to the gold surface (see Figure 21B, red representation) or as anchor points for the aptamers (green representation). Two different configurations were investigated: the ssDNA modification at the distal end (DE) or the lateral surface (LS) of the bundles. The ssDNA hybridized either to corresponding thiolated ssDNA that immobilize the bundles to the gold surface, or to a thrombin-binding aptamer as a model system, according to their specification. With this setup, the designed DNA nanostructures allowed the attachment of aptamers with nanoscale precision and predictable orientation.

For performance evaluation of the sensors, electrodes functionalized with tetrahedron structures as anchors for the aptamers were used as a reference. Compared to the tetrahedron-based sensor, the bundle-based yielded a wider linear range in the detection of thrombin, whereas the limit of detection were in the same range (tetrahedron: 10.7 nM, LS bundle: 11.2 nM, DE bundle: 6.1 nM). The functionalization of the gold surface was highly reproducible irrespective of the DNA nanostructures used. Therefore, the utilization of 24-helix bundles as anchor platform for the immobilization of aptamers represents a suitable alternative to tetrahedron structures.

### 5.3. Immobilization via Reduced Graphene Oxide, Pyrene, and Pyridine.

Graphene-based nanocomposites have gained increasing attention due to their intriguing properties, such as highly tunable surface area, outstanding electrical conductivity, good chemical stability, and excellent mechanical behavior, as well as their low cost. Via electrodeposition, reduced graphene oxide can be grafted onto gold electrodes and function as immobilization platforms for bioreceptors as well as for antifouling agents such as PEG. Available options and recently utilized approaches for either covalent attachments or π–π interactions with aromatic structures will be presented in this section. These can easily be mixed and matched and therefore offer a plethora of opportunities for sensor-surface functionalization. Furthermore, amplification strategies that rely on the binding of graphene oxide nanosheets to aptamers are discussed, which emphasize the inherent antifouling properties of sandwich-like approaches that only amplify signals derived from the sensors’ interaction with the analyte, while signals of possible fouling remain unamplified. 

#### 5.3.1. Reduced Graphene Oxide Deposits as Interface for Covalent Aptamer Immobilization

Graphene can be applied to gold surfaces via the electrophoretic deposition (EPD) of graphene oxide (GO) to introduce a platform for a large variety of subsequent functionalizations. In this process, charged GO particles migrate under the influence of an electric field towards the oppositely charged electrode where they deposit, and form reduced graphene oxide (rGO) films. In this process, graphene oxide is never reduced completely to form a perfect graphene layer; some oxygen-containing functional groups always remain [143]. 

**Figure 22:**
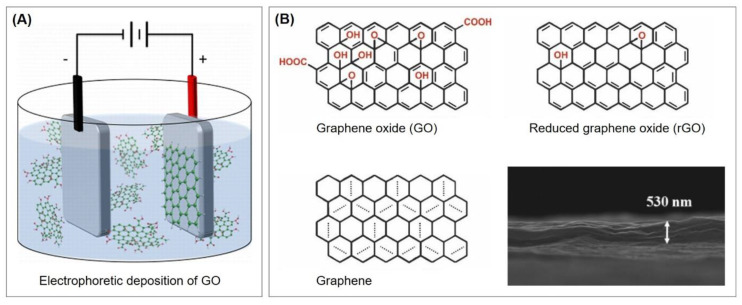
(**A**) Schematic representation of electrophoretic deposition, reproduced with permission from the authors of [159], copyright 2013, American Chemical Society. (**B**) Structures of graphene oxide (GO), reduced graphene oxide (rGO), and graphene, reproduced from the paper [143]. Cross-sectional FE-SEM image of deposited rGO membrane in layer-by-layer morphology. Here, EPD was performed at RT and the resulting membrane was detached from the substrate by electrochemical etching. Reproduced with permission from the authors of [160], copyright 2014, American Chemical Society.

Depending on the charge of GO, it is either attracted to the anode or the cathode. Graphene oxide, as pictured in Figure 22B, exhibits a number of carboxyl and hydroxyl groups, which result in a negative net charge and account for the migration to the anode. Anionic EPD, unfortunately, does not reduce GO to the desired extend, which is why an addition thermal or chemical reduction step is necessary to obtain a highly conductive material [144]. To allow the cathodic EPD, metal ions, such as Ni^2+^ [145], Cu^2+^ [146], and Mg^2+^ [147], are utilized that adsorb to GO and account for a positive net charge. Alternatively, GO is combined with positively charged polymers that result in the deposition of nanocomposites, which is furthermore advantageous for the subsequent integration of surface ligands [144]. To name a few examples, polyethyleneimine [23,148,149,150], polyaniline [151,152], poly(o-anisidine) [153], polypyrrole [154], and chitosan [155] have been employed.

In order to obtain a densely packed layer, a stable GO suspension is required, in which the individual graphene particles do not agglomerate and move independently, which enables their rearrangement during deposition [156]. For this purpose, the particle net charge plays a crucial role. If the charge is too low, the particles coagulate, which results in porous, sponge-like deposits, whereas charges that are too high lead to particle repulsion. During deposition, the particles come in close contact via attraction forces to form cohesive and dense layers [157]. This requires a charge loss and rearrangement, whereas particle/electrode reactions are not involved. The exact mechanisms that are taking place during deposition are still not entirely clear. 

For the fabrication of aptasensors, a plethora of opportunities for the sensor-surface functionalization is available. Covalent surface modifications of graphene-based materials can be easily realized by the use of diazonium salts. This strategy is a popular approach, because diazonium salts allow the introduction of different chemical groups, such as −B(OH)_2_, −COOH, and −C≡CH [24,144]. After the release of N_2_ molecules and the generation of radicals, diazonium salts form covalent bonds with the sp_2_ hybridized carbon lattice atoms of rGO films [24]. Amidation or esterification of the carboxyl groups present in rGO is also widely employed. The previously activated carboxylic groups thereby react with a primary amine or a primary alcohol as a nucleophilic partner to form amide or ester bonds [158]. If the electrophoretic deposition took place in combination with a polymer to form a nanocomposite, the polymeric compound can alternatively act as basis for the introduction of surface ligands, which opens an enormous variety of modification opportunities. 

An elegant alternative to covalent linkage lies in the noncovalent functionalization of the rGO deposit via π–π interactions with aromatic structures. Pyrene, a polycyclic aromatic hydrocarbon consisting of four fused benzene rings, is intensely investigated as a linker for the immobilization of different bioreceptors [161,162,163,164]. To minimize nonspecific adsorption, which is prominent on graphene, the modification with pyrene-polyethylene glycol has been shown to be the best antifouling strategy [23,24,148,162,165,166,167]. As an alternative aromatic linker, porphyrins are utilized [168] that are composed of four modified and interconnected pyrrole subunits and are best known for their role as basis for heme and chlorophyll. 

**Figure 23:**
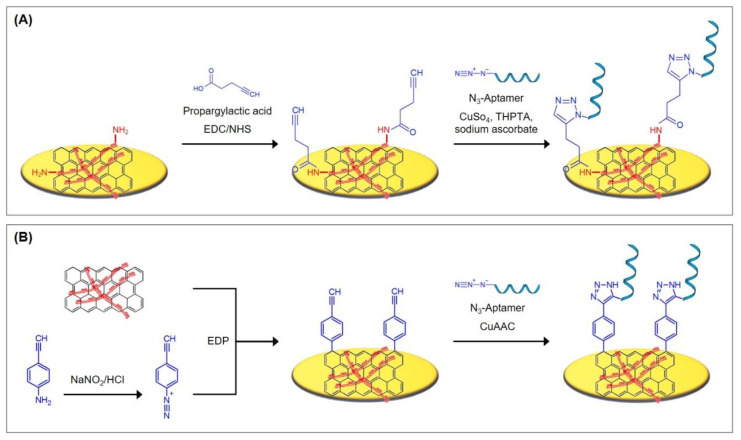
Various recently employed immobilization strategies based on the electrodeposition of graphene oxide in combination with PEI: (**A**) Immobilization of azide-terminated aptamers onto PEI [23]; (**B**) Immobilization of azide-terminated aptamers onto rGO [24].

Recently, the electrophoretic deposition of polyethyleneimine (PEI)/reduced graphene oxide (rGO) films was employed by Grabowska et al. [23] for the detection of natriuretic peptide (BNP-32), a cardiovascular biomarker for acute heart failure. PEI exhibits primary amines at the chain ends, which can be covalently linked to the carboxyl groups present in GO through a carbodiimide cross-linking reaction. The presence of NH_2_ groups in branched PEI also enables the subsequent covalent linking of ligands, in this case propargylactic acid via EDC/NHS chemistry, to which azide-terminated aptamers can be immobilized using Cu(I)-based click chemistry (see Figure 23A and Protocol A11). Finally, the aptasensor was incubated in pyrene-PEG, which interacts with the rGO deposit via π–π interactions and inhibits biofouling.

The combination of GO and PEI allowed the cathodic electrophoretic deposition of a homogenous, well-reduced graphene oxide film that increases the surface roughness and the electrochemically active surface area of the electrode. The coating results in an increased anionic peak current, which is due to the good electronic properties of the rGO/PEI film, enhanced surface area, and favorable electrostatic interactions between the negatively charged redox mediator and the positively charged rGO/PEI interface. 

The BNP aptasensor exhibited excellent analytical performance with a wide linear range between 1 pg/mL and 1 µg/mL and a detection limit of about 0.9 pg/mL in differential pulse voltammetry. The serum levels of healthy patients (< 35 pg/mL [169]) and of those at high risk of heart failure (> 500 pg/mL [169]) both fit in the linear range of the sensor so that prior dilution of patient serum samples is neither necessary nor possible. Biofouling was therefore tested with appropriate concentrations of myoglobin (test: 1 mg/mL, healthy serum level: 0–95 ng/mL) and albumin (test: 60 mg/mL, healthy serum level: 35–50 mg/mL), which did not alter the resulting electrochemical signal, demonstrating the exceptional antifouling properties of the aptasensor. Accordingly, when tested with spiked serum samples, the aptasensor demonstrated excellent recoveries ranging 98%–108%. Taking the remarkable reproducibility (tested with 10 electrodes that showed 5.9% signal variation) into account, the investigated BNP aptasensor, which is based on the electrophoretic deposition of a rGO/PEI film with subsequent aptamer immobilization and blocking with pyrene-PEG, demonstrates outstanding accuracy and reliability. 

Wang et al. [24] followed a similar approach in performing the electrophoretic deposition of GO in combination with PEI, but decided to immobilize the aptamer via a diazonium linker that bears an alkynyl function and is covalently bound to rGO. The aptasensor was designed to detect lysozyme, which is used as a biomarker for Inflammatory Bowel Disease (IBD), where lysozyme levels are up-regulated. The nanocomposite formation of GO and PEI with diazonium functionalization took place in a single step by simultaneous electrophoretic deposition. PEI and GO cross-link covalently by the carbodiimide reaction of primary amines present in PEI and carboxylic groups present in GO. With the application of a DC potential in the presence of NaNO_2_/HCl, the diazonium precursor, namely 4-ethynylaniline, forms a salt, followed by elimination of nitrogen and reduction to an aryl radical that builds a covalent bond with rGO. XPS analysis confirmed the covalent coupling, since the overall nitrogen content of the rGO/PEI/diazonium deposit is the same as of rGO/PEI alone, indicating that 4-ethynylaniline did not bind to graphene via π–π stacking. The obtained nanocomposite is a homogeneous film of about 1 nm thickness that almost entirely blocks the diffusion of redox mediators to the electrode surface, which is why cyclic voltammetry was found to be unsuitable and differential pulse voltammetry was chosen for evaluation. 

Azide-terminated aptamers were bound to the alkynyl functional groups of the diazonium linker in a copper-catalyzed azide–alkyne cycloaddition reaction (CuAAC), which generates a triazole functionality that links the aptamer to the nanocomposite (see Figre 23 B and Protocol A12). The rGO/PEI/4-ethynylaniline deposit therefore represents a generic interface that allows the convenient immobilization of azide-terminated aptamers of interest by simple click chemistry. The aptasensor exhibited a remarkably low detection limit of 200 fM with a linear range up to 30 pM without the employment of any amplification technique. If 4-ethynylaniline is directly linked to the gold electrode without the incorporation of rGO and PEI, the resulting aptasensor shows a linear range of 1–15 pM, which points at the high aptamer density on the rGO/PEI/4-ethynylaniline nanocomposite and the sensors sensitivity. Since the aptasensor is passivated with BSA prior to use, the changes in currents were insignificant when treated with BSA, cytochrome C or casein for the investigation of antifouling properties. Serum samples of healthy people and of patients suffering from IBD before and after treatment were tested and the resulting lysozyme concentrations were found to be analogous to those obtained by a classis turbidimetric assay. Therefore, the investigated aptasensor demonstrated outstanding accuracy and reliability and is adequate for the analysis of clinical samples.

#### 5.3.2. Aptamer Immobilization via Non-Covalent π–π Interactions of Graphene, Pyrene, and Porphyrin

**Figure 24:**
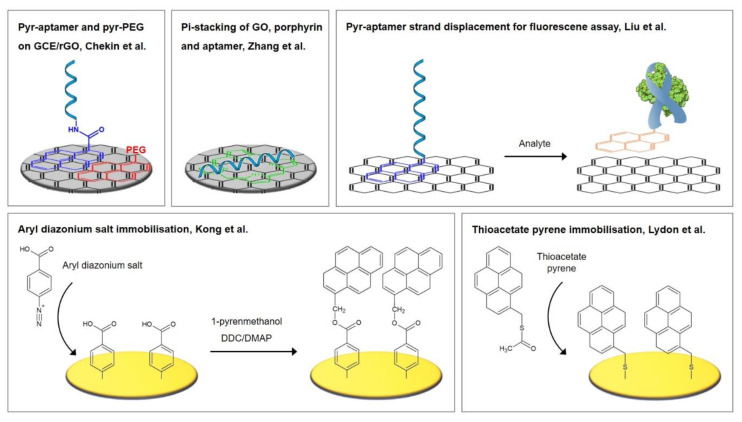
Schematic presentation of possible strategies incorporating π–π interactions with pyrene for aptamer immobilization [22,162,163,170,173,174].

An elegant alternative to covalent linkage lies in the noncovalent functionalization of the rGO deposit via π–π interactions with aromatic structures like pyrene and porphyrin that carry the bioreceptors. In the same way, pyrene-labeled PEG can be bound to the rGO interface to provide a biosensor with excellent antifouling properties [23,24,148,162,165,166,167]. The noncovalent attachment of aromatic complexes does not strictly rely on the utilization of graphene; instead, gold surfaces can also be pre-functionalized with pyrene molecules, e.g., via Au–S chemistry [170] or diazonium salts [22], to represent the interface for aptamer immobilization. Since any aromatic structures with delocalized π electrons can interact with each other by π–π stacking and can be individually bound to gold and electrodes of other materials, the application of graphene, pyrene, and porphyrin offers a plethora of opportunities for sensor-surface functionalization. To the best of our knowledge, no research articles that fit into the framework of our review and utilize π–π interactions of aromatic structures have been published in the last three years. Nevertheless, we want to discuss relevant articles of this topic to demonstrate promising strategies and refer the reader to the corresponding literature. The following ideas are summarized in Figure 24 and can easily be mixed and matched, and therefore offer an interesting outlook for future research in the field of aptasensors. 

Chekin et al. [162] offer a protocol to link aminated aptamers to 1-pyrenecarboxylic acid (py-COOH) via EDC/NHS chemistry. In a step-by-step process, they produced nitrogen-doped porous reduced graphene oxide (N-prGO) to immobilize it on glassy carbon electrodes, which were further noncovalently modified by py-COOH with subsequent binding of the Tro4 aptamer. Poly(ethylene glycol) modified pyrene (py-PEG) was also synthesized following a protocol from the work of [171] and bound to the N-prGO interface to inhibit biofouling. The aptasensor was designed for the detection of cardiac troponin I, a cardiac protein biomarker. The sensor demonstrated an exceptionally low detection limit of 0.88 pg/mL and a wide linear range over 6 orders of magnitude with 1 pg/mL to 100 ng/mL in differential pulse voltammetry. When the sensor was tested with lysozyme, BSA, and BNP (brain natriuretic peptide) in concentrations that correspond to their undiluted serum levels, the sensor demonstrated its excellent antifouling properties, since no significant change in current density could be observed. 

Liu et al. [163] also linked aptamers to pyrene that furthermore absorbed on the surface of GO, which built the basis for their fluorometric assay for cardiac myoglobin detection, a protein biomarker for myocardial infarction. Aromatic hydrocarbons like pyrene exhibit fluorescence when their concentration exceeds 100 mol/l and when no acceptor is present to which pyrene can bind [172]. In the absence of myoglobin, py-aptamer binds to GO, which quenches the fluorescence by FRET mechanism so that no signal can be detected. If myoglobin is added, it builds a complex with py-aptamer that thereupon dissociates from GO. Therefore, with increasing target concentration, an increasing fluorescence intensity can be measured. The sensor exhibited a detection limit of 3.9 pM and a linear range of 5.6 pM–449.4 pM and did not show remarkable interference from other substances. Since the aptamer has a high affinity towards myoglobin, only the target molecule can cause dissociation and trigger fluorescence. 

As an alternative to the interaction with carbon-based surfaces, gold electrodes can be pre-functionalized with covalently bound pyrene, which represents the interface for aptamer immobilization via π–π stacking. For this purpose, Lydon et al. [170,175] synthesized thioacetate functionalized pyrene (S-(pyren-1-ylmethyl) ethanethioate) that can be covalently bound to gold via Au–S chemistry. The product can be isolated by nucleophilic substitution of 1-bromomethylpyrene with potassium thioacetate and has to be deprotected prior to its attachment onto gold by a strong base, e.g., NH_4_OH. In contrast to their thiol counterparts, thioacetate derivatives are not prone to oxidation to form disulfides or sulfoxides, which makes them easier to isolate and purify. 

The working group investigated the electron transfer through the pyrene–pyrene interface with the help of 1-pyrenylferrocene and came to the conclusion that this hybrid noncovalent attachment of the redox mediator demonstrates an approximately ten times higher electron transfer rate constant compared to covalent ferrocene attachment. Furthermore, the pyrene-functionalized ferrocene is stable on the surface even after washing with solvents in which the molecular species is soluble.

Kong et al. [22] modified a gold electrode with aryl diazonium salts by an electrochemical reductive method: the electrode was immersed in an aqueous solution of the salt and cyclic voltammetry was performed with a scan rate of 100 mV/s for three cycles between +1.0 and −1.0 V. This way, a surface-to-carbon bond is formed via the one-electron reductive formation of an aryl radical, which subsequently attacks the electrode surface. To the carboxylic groups, pyrenemethanol was subsequently attached through esterification coupling in the presence of N,N-dicyclohexyl carbodiimide and 4-dimethylaminopyidine. The surface coverage of pyrene groups was calculated to be 1.23 × 10^11^ mol/cm^2^, which led to the conclusion that not all carboxylic groups were functionalized, which XPS spectra confirmed. The aryl diazonium salt, namely 4-carboxyphenyl diazonium tetrafluoroborate salt, was synthesized from 4-aminobenzoic acid according to a protocol from the literature [176,177]. The immobilized pyrene molecules were used to bind graphene nanosheets via π–π stacking. To these, lead and copper ions specifically adsorb and were detected via Osteryoung square wave voltammetry (OSWV). 

Zhang et al. [173,174] fabricated a thrombin aptasensor that was solely based on the noncovalent interactions of its components. A graphene oxide/porphyrin nanocomposite was cast onto a glassy carbon electrode with subsequent addition of the aptamer, which bound to the nanocomposite via π–π interactions. Porphyrin exhibits excellent electrochemical activity and can therefore substitute the redox mediator. The porphyrin π-ring system undergoes a one-electron transfer to form a cation/anion radical that can be converted to its dicationic/dianionic form at more positive/negative potentials [178]. The aptamer immobilization reduces the electron transfer of porphyrin, which is further hindered by thrombin binding, resulting in decreased signals in differential pulse voltammetry. The proposed aptasensor is simple and robust, and benefits from the synergetic effects of its components: the high conductivity and large specific surface area of graphene, the excellent electrochemical activity of porphyrin, and the high affinity and specificity of the aptamer. It exhibits a detection limit of 200 pM and a linear range of 5 nM–1.5 µM, and low nonspecific interaction with BSA and HSA.

## 6. Concluding Remarks and Future Perspectives

In this review, we have discussed recently published (2017–2019) immobilization techniques for aptamers on gold electrodes for the electrochemical detection of proteins. Our focus rested on the detailed discussion of underlying principles and the presentation of utilized chemical protocols to provide the reader with promising ideas and profound knowledge of the subject, as well as an update on recent discoveries and achievements.

Simple and straightforward immobilization strategies were reviewed that either rely on the direct immobilization of thiolated aptamers or on the utilization of short, thiolated linkers, such as cysteamine, 3-mercaptopropionic acid, 3,3′-dithiodipropionic acid, Lomant’s Reagent, and aromatic thiols. The aptamers are oftentimes immobilized in a mixed self-assembled monolayer via co-immobilization or backfilling with short alkanethiols, preferably mercaptohexanol. This approach aims to prevent unspecific interactions of aptamers with the gold surface, ensuring their upright orientation and dense packing, as well as blocking of unoccupied gold surface to inhibit fouling. Nevertheless, the utilization of mercaptohexanol faces a number of serious drawbacks that can result in the irreproducibility of the fabrication, impaired target binding, as well as nonspecific binding of interfering molecules that lead to false responses. 

A number of different strategies have previously been investigated to overcome biofouling. However, the majority of these techniques incorporate high-molecular-weight compounds, which are highly disadvantageous for electrochemical transfer reactions. Therefore, only a small number of antifouling strategies exists that are compatible with electrochemical measurements. In general, PEG or zwitterionic SAMs are considered the most efficient approaches to inhibit fouling. In the framework of this review, aromatic thiols, adamantane molecules, and DNA nanostructures, such as tetrahedrons and helix bundles, as linkers or immobilization platforms for aptamers, as well as zwitterionic peptides, have demonstrated their promising antifouling properties. 

For the detection of low abundance analytes, signal amplification is often necessary. Widely employed are strategies that aim to increase the specific surface area, which enables the immobilization of a greater number of aptamers. For this purpose, gold nanoparticles were either directly attached to the electrode via electrodeposition or bound to a prior immobilized alkanethiol SAM. Immobilized dendrimers also offer a vast number of functional end groups for the attachment of aptamers. Binding of the redox mediator, either covalently or via intercalation, is also commonly used. Strategies based on the elongation of the aptamers were discussed, namely hybridization chain reaction or rolling circle amplification. Finally, a newly introduced strategy, the target-induced bridge assembly, was presented, which applies two different aptamers for the same target. 

The last section focused on graphene-based strategies, which also offer an interesting outlook for future investigations, since they can easily be mixed and matched: via electrodeposition, reduced graphene oxide can be grafted onto gold electrodes and function as immobilization platforms for bioreceptors as well as for antifouling agents such as PEG. Available options and recently utilized approaches for either covalent attachments or π–π interactions with aromatic structures are discussed and complemented by graphene oxide nanosheet based amplification strategies. These emphasize the inherent antifouling properties of sandwich-like approaches that only amplify signals derived from the sensors’ interaction with the analyte, while signals of possible fouling remain unamplified. 

Future investigations should aim to investigate reliable sensing interfaces that are highly reproducible, stable, and able to even detect low-abundance proteins, for which a variety of amplification strategies are already available. Efforts have to be made to establish suitable and stable antifouling strategies that prove their applicability in complex matrices, such as whole blood, serum, urine, and saliva, to meet their requirements for commercialization and clinical applications.

## Figures and Tables

**Table 1:** Aptasensors that were fabricated by co-immobilization/backfilling of thiolated aptamers with alkanethiols.

Target	Aptamer Modifi-cation	Fabrication Procedure	Method	LOD	Linear Range	Ref
Protein tyrosine kinase-7 (PRK7) on leukemic Jurkat cells	5′-thiol	Co-immobilization with MCH	EIS	105 cells/mL	50–500.000 cells/mL	[31]
C-reactive protein (CRP)	5′-thiol-C_6_	Backfilling with MCH	CV	1 pM	1–100 pM	[32]
HER-2/neu breast cancer biomarker	5′-thiol-C_6_	Backfilling with SH-C_11_-(EG)_2_-OH	CV	1 pM	1 pM–10 nM	[33]
Estrogen receptor alpha (ERα)	5′-thiol-C_6_	Only aptamer	DPV	15 fM	15 fM–15 nM	[34]
Plasmodium falciparum glutamate dehydro-genase (PfGDH)	5‘-thiol-C_6_	Co-immobilization and backfilling with MCH	EIS	430 fM	100 fM–100 nM	[35]
Protein A on Staphylococcus aureus	3′-thiol-C_6_	Co-immobilization and backfilling with MCH	EIS	10 CFU/mL	N/A	[36]

**Table 2:** Most abundant proteins in human serum [67,68,69].

Protein	Serum Concentration	Protein	Serum Concentration
Albumin	35–50 mg/mL	Immunoglobulins	
Globulin	18–35 mg/mL	● IgA	● 0.7–4 mg/mL
Fibrinogen	2–4 mg/mL	● IgE	● 0.03–4 mg/mL
Haptoglobin	0.3–2 mg/mL	● IgG	● 7–16 mg/mL
Serum cholesterol	Total < 2 mg/mL	● IgM	● 0.5–2.5 mg/mL
● LDL	● < 1.3 mg/mL	● IgD	● 0–0.08 mg/mL
● HDL	● 0.45 mg/mL	Transferrin	0.17–0.37 mg/mL
● Triglyceride	● < 1.5 mg/mL	Hemoglobin	≤0.05 mg/mL

## Appendix A

In the following section, employed protocols of promising investigations that the reader was referred to throughout the review will be presented.

**Protocol A1 by Ciu et al. [77]: Mixed monolayer of aptamer and zwitterionic peptide** *Reagents* - 0.2 M PBS buffer pH 7.4; - 0.5 µM thiolated aptamer in PBS buffer; - 25 µM peptide in PBS buffer. *Method* 1. Incubate clean electrode in aptamer solution for 12 h at RT; 2. Incubate in peptide solution for 12 h at RT; 3. Wash extensively with PBS buffer; 4. Incubate in PBS buffer for 24 h at RT; For the bioassay, alpha-fetoprotein (AFP) was prepared in 10 mM PBS buffer pH 7.4. Linear concentration range: 10 fg/mL–100 pg/mL.	**Protocol A2 by Peng et al. [110]: Signal amplification via graphene oxide and methylene blue binding** *Reagents* - PBS buffer; - 2 mM mercaptohexanol (MCH) solution; - 0.5 µM thiolated aptamer solution in 25 mM Tris-HCl buffer containing 20 mM NaCl; - Bacteria solutions of differing concentrations (linear range: 2 × 10^1^ to 2 × 10^6^ CFU/mL) in PBS; - 1 mg/mL graphene oxide (GO) solution in PBS; - 120 µM methylene blue (MB) solution. *Method* 1. Incubate the cleaned electrode in aptamer solution for 5 h at 24 °C; 2. Rinse with PBS buffer; 3. Incubate in MCH solution for 1 h. For the bioassay, incubate in bacteria solution for 1 h, followed by subsequent incubation in GO solution for 4 h and MB solution for another 1 h.

**Protocol A3 by Jolly et al. [78]: Spherical AuNPs on 11-amino-1-undecanethiol SAM** *Reagents* - 1 mM 11-amino-1-undecanethiol (AUT) dissolved in pure ethanol; - 1 mM mercaptohexanol (MCH); - gold nanoparticles (20 nm, stabilized suspension in 0.1 mM PBS, reactant free); - aptamer solution: thiolated aptamer and MCH (ratio 1:50) in 10 mM PBS, pH 7.4. Prior to preparation, heat up the aptamers to 95 °C for 10 min and gradually cool down to RT. *Method* 1. Immerse the cleaned electrode in the AUT solution and incubate for 16 h at 4 °C; 2. Wash with pure ethanol and MilliQ water 3. Incubate in MCH solution for 1 h at RT; 4. Incubate in AuNP solution in an inverted position overnight; 5. Incubate in aptamer solution for 2 h at RT; 6. Rinse with ultra-pure water. For the bioassay, proteins were dissolved in 10 mM PBS, pH 7.4.	**Protocol A4 by Daems et al. [141]: 3D DNA tetrahedron as anchor for aptamer immobilization** *Reagents* - 1 µM stock solution of each oligonucleotide; - TM buffer: 20 mM Tris base, 50 mM MgCl_2_, pH 8.0; - 3 mM Tris-(2-carboxyethyl)-phosphin (TCEP); - TGK buffer: 25 mM Tris base, 192 mM glycine, 5 mM K_2_HPO_4_, 0.1% Tween 20 and 0.15 w/v% BSA, pH 8.3. *Method* 1. Create the tetrahedron solution by mixing 164 µL of TM buffer and 20 µL of TCEP with 4 µL of each oligonucleotide solution; 2. Heat to 95 °C for 2 min and cool to 4 °C (ramp speed −0.5 °C/min); 3. Incubate clean electrode with 200 µL tetrahedron mixture at 4 °C overnight; 4. Wash three times with TGK buffer; 5. For the bioassay, immerse the electrode in 120 µL TGK buffer for 5 min to reach a stable baseline. Immerse in thrombin solution (concentrations ranging 15.5–248 nM were investigated) in TGK buffer for 20 min, followed by a washing step of 3 min in TGK buffer.

**Protocol A5 by Negahdary et al. [80]: Electrodeposition of fern-leaf-like gold nanostructures with increased surface area for aptamer immobilization** *Reagents* - Solution for electrodeposition: 0.5 M H_2_SO_4_, 20 mM HAuCl_4_, 0.1 M polyethylene glycol (PEG) 6000; - 10 µM tris[2-carboxyethyl] phosphine; - Ethyl acetate; - 10 mM phosphate buffer, containing 5 mM NaCl, 2 mM KCl, 1 mM MgCl_2_, pH = 7.4; - 1 mM mercaptohexanol (MCH); - Artificial CSF: 126 mM NaCl, 2.5 mM KCl, 1.24 mM NaH_2_PO_4_, 1.2 mM MgSO_4_, 2 mM CaCl_2_, 26 mM NaHCO_3_, 10 mM D-glucose, pH 7.35. *Method* 1. Immerse the cleaned electrode in the corresponding solution. Electrodeposition was potentiostatically performed at 0 mV for a duration of 5 min; 2. Mix the thiolated aptamer (concentration? Amount?) with 3.6 µL of 10 µM tris[2-carboxyethyl] phosphine and vortex for 2 h; 3. Add ethyl acetate in three portions (total volume of 100 μL), and remove the upper layer after each addition; 4. Mix the resultant liquid with the phosphate buffer solution; 5. Incubate the electrode in the obtained solution for 1 h at 4 °C; 6. Rinse with deionized water; 7. Incubate with 1 mM MCH solution for 30 min at RT; 8. Rinse thoroughly with deionized water. For storage, incubate in 20 mM Tris-HCl buffer at 4 °C. For the bioassay, incubate with Aβ (linear range: 2 pg/mL–1.28 ng/mL, diluted in artificial CSF) for 10 min at 37 °C and rinse with deionized water. For regeneration, immerse in deionized water for 5 min at 95 °C to release bound Aβ.

**Protocol A6 by Malvano et al. [42]: Cysteamine, glutaraldehyde, and PAMAM dendrimer scaffold** *Reagents* - 20 mM cysteamine solution; - 5% (v/v) glutaraldehyde solution; - Activation solution: 75 mM N-(3-dimethylaminopropyl)-N-ethylcarbodiimide hydrochloride (EDC) and 15 mM N-hydroxysuccinimide (NHS) in MES buffer; - MES buffer: 100 mM 2-(N-morpholino) ethanesulfonic acid, pH 7.4; - PAMAM solution (1 mg/mL, 1.5 mg/mL, and 2 mg/mL were investigated, highest sensitivity with 2 mg/mL); - 0.5 µM aptamer solution; - Phosphate buffer saline (PBS) solution: 137 mM NaCl, 2.7 mM KCl, 8.1 mM Na_2_HPO_4_ and 1.47 mM KH_2_PO_4_, pH 7.5. *Method* 1. Drop the cysteamine solution on the cleaned electrode and apply a constant potential of 1.2 V vs. Ag/AgCl for 20 min; 2. Thoroughly rinse with water; 3. Drop 100 µL of glutaraldehyde solution on the electrode and incubate for 1 h; 4. Thoroughly rinse with water; 5. Incubate with the PAMAM solution for 3 h; 6. Treat the aptamer with activation solution for 2 h; 7. Incubate the electrode with the aptamer solution; 8. Rinse with PBS buffer.

**Protocol A7 by Cao et al. [13]: Dual-signaling strategy of aptamer-labelling with ferrocene and the intercalation of RuHex** *Reagents* - 100 mM Tris-HCl buffer containing 100 mM NaCl and 10 mM TCEP, pH 7.4; - 20 µM A-Lys solution in 100 mM Tris-HCl buffer, containing 100 mM NaCl, pH 7.4; - Washing buffer: 10 mM Tris-HCl, pH 7.4; - 1 mM mercaptohexanol (MCH) solution in 10 mM Tris-HCl; - Lysozyme solutions (linear range: 10 pM to 100 nM) in 100 mM Tris-HCl, containing 100 mM NaCl, pH 7.4; - High ionic strength solution: 100 mM Tris-HCl containing 140 mM NaCl and 5 mM MgCl_2_, pH 7.4; - Detection solution: 100 mM Tris-HCl containing 10 µM RuHex, 140 mM NaCl and 5 mM MgCl_2_, pH 7.4; - Regeneration solution: 1 µM A-Lys solution in 100 mM Tris–HCl, containing 100 mM NaCl, 1 mM MgCl_2_, pH 7.4. *Method* 1. Prepare a 20 µM P-Fc solution in Tris/NaCl/TCEP buffer and incubate for 1 h at RT; 2. Mix the P-Fc and A-Lys solutions and dilute to obtain 2 µM for each ssDNA; 3. Heat to 90 °C for 5 min, gradually cool down to RT, and incubate at 37 °C for 2 h; 4. Incubate the cleaned electrode in the DNA solution for 20 h at RT; 5. Rinse with washing buffer; 6. Incubate with MCH solution for 1 h; 7. Rinse with double-distilled deionized water and washing buffer in sequence. For the bioassay: 1. Incubate with lysozyme solution for 70 min at 37 °C; 2. Rinse with washing buffer; 3. Immerse in the high ionic strength solution for 30 min at RT; 4. Immerse in detection solution for 6 min. For regeneration, immerse the sensor in regeneration solution for 2 h. Rinse with double-distilled deionized water and washing buffer in sequence.

**Protocol A8 by Ding et al. [105]: Adamantane as surface anchor for aptamers and signal amplification via amplification chain reaction** *Reagents* - 7-hydroxycarbonyl-2,4,9-trithiaadamantane was synthesized according to a previous report [179]. - Adamantane solution consisting of 1 µM 7-hydroxycarbonyl-2,4,9-trithiaadamantane, 10 mM Tris-HCl, 1 mM EDTA, 10 mM TECP; - Carboxyl group activation solution consisting of 200 mM EDC and 50 mM NHS; - 1 µM capture probe solution; - 4 µM mix of H1 probe and H2 probe; - 10 mM phosphate buffer, pH 7.4; - Analyte solution: PSA in phosphate buffer, containing 0.25 M NaCl. PSA concentrations ranging 0.1 pg/mL–0.1 µg/mL were investigated. *Method* 1. Immerse the cleaned electrode in the adamantane solution for 4 h; 2. Incubate with carboxyl group activation solution for 30 min; 3. Rinse with double-distilled water; 4. Incubate with capture probe solution for 1 h; 5. Incubate with analyte solution for 1 h, followed by careful rinsing; 6. Incubate with H1&H2 probe solution for 2 h.

**Protocol A9 by Meirinho et al. [120]: Immobilization of biotinylated aptamer via streptavidin** *Reagents* - 200 mM 3,3′-dithiodipropionic acid (DPA) solution; - 200 mM N-(3-dimethylaminopropyl)-N-ethyl-carbodiimide hydrochloride (EDC) solution; - 100 mM N-hydroxysuccinimide (NHS) solution; - 100 mM, pH 8.5 ethanolamine (ETA) solution; - Phosphate buffer saline (PBS) solution (137 mM NaCl, 2.7 mM KCl, 8.1 mM Na_2_HPO_4_ and 1.47 mM KH_2_PO_4_), pH 7.4; - 4 nM biotinylated aptamer solution in PBS buffer. *Method* 1. Incubate cleaned electrode with DPA solution for 30 min at RT and then rinse with deionized water; 2. Treat with EDC and NHS solutions (1:1 v/v) for 1h at RT; 3. Incubate with streptavidin solution overnight at 4 °C; 4. Treat with ETA for 20 min at RT; 5. Heat up the aptamer solution to 95 °C for 5 min, cool down to 4 °C for 5 min and at RT for 10 min; 6. Place 5 µL aptamer solution on the electrode for 40 min at RT and rinse with PBS buffer.

**Protocol A10 by Daems et al. [141]: Immobilization of 24-helix DNA bundle as anchor platform for aptamer immobilization** *Reagents* - PEG 8000 precipitation buffer: 5 mM Tris base, 1 mM Na_2_EDTA, 505 mM NaCl, 15 w/v% PEG 8000; - TEMg buffer: 5 mM Tris base, 1 mM Na_2_EDTA, 13 mM MgCl_2_, pH 7.6; - Phosphate buffer: 12 mM K_2_HPO_4_ and 167 mM K_2_HPO_4_, pH 8.3; - TNM buffer: 10 mM Tris base, 25 mM MgCl_2_, 2 M NaCl, pH 8.0; - TGK: 25 mM Tris base, 192 mM glycine, 5 mM K_2_HPO_4_, 0.1% Tween 20 and 0.15 w/v% BSA, pH 8.3; - 0.01% sodium dodecyl sulfate (SDS). *Method for the one-step assembly and purification (underlined steps are described in Stahl et al. [180])* 1. Prepare the self-assembly reaction mixture, containing 20 nM aptamer, 200 nM of each staple DNA, 5 mM TRIS, 1 mM EDTA, 20 mM MgCl_2_ and 5 mM NaCl (pH 8); 2. Incubate the reaction mixture at 65 °C for 15 min, anneal from 60 °C to 40 °C in steps of 1 °C per 2–3 h; 3. Mix with the PEG 8000 precipitation buffer in a 1:1 (v/v) ratio and adjust to 20 mM MgCl_2_; 4. Mix the solution by tube inversion and centrifuge at 16,000× *g* for 25 min at RT; 5. Remove the supernatant and dissolve the pellet in TEMg buffer for 30 min at 30 °C; 6. For the spin column purification, wash the Amicon Ultra 0.5 mL centrifugal filter devices with 500 µL of TEMg (containing 6 mM MgCl_2_) by centrifugation at 10,000× *g* for 5 min; 7. Add 50 µL of the prepared DNA sample to the device together with 450 µL TEMg and centrifuge at 4500 *g* for 5 min; 8. Remove the excess of staple strands and recover the nanostructures by reverse spinning of the centrifugal filter device. *Method for immobilization* 1. Activate the thiolated ssDNA linkers with 0.1 M dithiothreitol that is removed with a NAP-5 column afterwards; 2. Incubate the sensor in 1 µM activated thiolated ssDNA, dissolved in phosphate buffer; 3. Wash three times in phosphate buffer with 0.01% SDS; 4. Incubate the sensor with the assembled DNA nanostructure with a concentration of 16 nM (LS) or 83 nM (DE) in TNM buffer at 4 °C overnight; 5. Wash with TGK; 6. For the bioassay, immerse the sensor in 120 µL TGK buffer for 5 min to reach a stable baseline; 7. Immerse in thrombin solution (concentrations ranging 15.5–248 nM were investigated) in TGK buffer for 20 min, followed by a washing step of 3 min in TGK buffer.
**Protocol A11 by Grabowska et al. [23]: Electrophoretic deposition of rGO/PEI nanocomposite and immobilization of azide-terminated aptamers onto PEI** *Reagents* - Polyethylenimine (PEI); - 1-Ethyl-3-(3-imethylaminopropyl)carbodiimide (EDC); - tris(3-hydroxypropyltriazolylmethyl)amine (THPTA); - N-Hydroxysuccinimide (NHS); - Propargylactic acid; - Copper(II) sulfate pentahydrate (CuSo_4_); - Sodium ascorbate; - 0.1 M phosphate buffer (PBS); - Pyrene-PEG, which can be synthesized following the method found in [148]. *Method* 1. Create GO/PEI dispersion by stirring graphene oxide powder and PEI, each 1 mg/mL, for 48 h at RT; 2. Immerse the SPGE in the GO/PEI dispersion and apply a DC voltage of 120 V for 20 s between the SPGE (cathode) and a Pt plate (anode); 3. Treat the azide-terminated apatamers with a mix of 25 mM EDC, 25 mM NHS, and 20 mM propargylactic acid for 2 h; 4. Incubate the electrode in a mix of 1 µM aptamer solution, 10 mM CuSO_4_, 100 mM sodium ascorbate, and 20 mM THPTA for 7 h at RT; 5. Wash the modified electrode three times with PBS; 6. Immerse the electrode in 1 mM pyrene-PEG for 2 h at RT. For the regeneration of the aptasensor, immerse in 0.1 M NaOH (pH 12) for 20 min.

**Protocol A12 by Wang et al. [24]: Electrophoretic deposition of rGO/PEI nanocomposite and immobilization of azide-terminated aptamers onto rGO** *Reagents* - Graphite powder (< 20 micron); - Polyethyleneimine (PEI); - Tris buffer; - 0.01 M phosphate buffer (PBS), pH 7.4; - Diazonium reagent mixture: 0.5 mL 15 mM diazonium reagent, 0.5 mL 15 mM NaNO_2_, 0.5 mL 0.75 M HCl; - Aptamer solution: 1 µM azide-terminated aptamer in 0.02 M Tris buffer, 1mM CuSo_4_, 1 mM sodium ascorbate, 2 mM tris(3-hydroxypropyl-triazolylmethyl)amine (THPTA). *Method* 1. Prepare the diazonium reagent mixture and stir at 0 °C for 5 min; 2. Add 2 mL of 0.5 mg/mL GO and 0.5 mL of 2 mg/mL PEI and apply a DC voltage of 15 V for 2 min; 3. Rinse three times with deionized water and blow dry with nitrogen gas; 4. Immerse the electrode in the aptamer solution at 4 °C overnight; 5. Wash with PBS; 6. Incubate in 5wt% BSA for 1 h; 7. Rinse with Tris buffer.
